# Disentangling Taxonomic Confusions in the *Aporia agathon* Group Using Mitochondrial Genomic Data (Lepidoptera: Pieridae)

**DOI:** 10.3390/insects15120988

**Published:** 2024-12-12

**Authors:** Shao-Ji Hu, Ya-Qi Jia, Xin Zhang, Yu-Feng Hsu, Alexander L. Monastyrskii, Van Lien Vu, Si-Xun Ge, Kuang Duan, Zhuo-Heng Jiang, Valerio Sbordoni, Min Wang

**Affiliations:** 1 Institute of International Rivers and Eco-Security, Yunnan University, Kunming 650500, China; jiayq_77@163.com (Y.-Q.J.); jzhsphingidae@163.com (Z.-H.J.); 2 Yunnan Key Laboratory of Institute of International Rivers and Transboundary Eco-Security, Yunnan University, Kunming 650500, China; 3 Kunming Youning Biotech Co., Ltd., Kunming 650051, China; monkey.z@163.com; 4 Department of Life Sciences, National Taiwan Normal University, Taipei 116, Taiwan, China; t43018@ntnu.edu.tw; 5 Vietnam National Museum of Nature, Vietnam Academy of Science and Technology, Ha Noi 10072, Vietnam; maack60@hotmail.com (A.L.M.); vulien@gmail.com (V.L.V.); 6 College of Forestry, Beijing Forestry University, Beijing 100091, China; 954236154g@sina.cn; 7 Institute of Resource Plants, Yunnan University, Kunming 650500, China; duankuang@ynu.edu.cn; 8 School of Science, Westlake University, Hangzhou 310024, China; 9 Dipartimento di Biologia, Universita di Roma, I-00133 Roma, Italy; valerio.sbordoni@uniroma2.it; 10 Department of Entomology, College of Plant Protection, South China Agricultural University, Guangzhou 510642, China

**Keywords:** black-vein pierids, type specimens, Sino-Himalayan species, Hengduan Mountains, new subspecies, phylogenetic reconstruction, male genitalia, female genitalia

## Abstract

Pierid species of the *Aporia agathon* group are among the largest Sino-Himalayan members of genus *Aporia*, with four conventionally recognised species, namely *A. agathon*, *A. largeteaui*, *A. gigantea*, and *A. lemoulti*. Recent publications indicated that some of these species may contain more than one species despite their similar morphological characters. The present research analysed this group of butterflies using mitogenomic data, and proved that *A. japfuensis* **stat. nov.**, *A. bifurcata* **stat. nov.**, *A. moltrechti* **reinst. stat.**, *A. kuangtungensis* **stat. nov.** and *A. omotoi* **stat. nov.** should be recognised as distinct species, while *lemoulti* **stat. rev.**, *gigantea* **stat. nov.** and *fanjinensis* **stat. rev.** should be subspecies of *A. largeteaui*. Two new subspecies, namely *A. kuangtungensis yufeii* **ssp. nov.** and *A. kuangtungensis josephi* **ssp. nov.** were described. Illustrations of specimens and male and female genitalia of each taxon were provided in this article with an updated classification system. Variable melanism and yellow tinge are two major factors causing historical taxonomic confusion; thus, such characters should be less crucial for this species group. Future research is still necessary to elucidate the evolutionary history of these species along with other *Aporia* taxa, as well as the mechanism of variable melanism and yellow coloration.

## Simple Summary

An updated classification system for the *Aporia agathon* group is proposed after the re-examination of a long series of specimens with mitogenomic data. Our results showed that *A. japfuensis*, *A. bifurcata*, *A. moltrechti*, *A. kuangtungensis*, and *A. omotoi* should be recognised as full species, while *lemoulti*, *gigantea*, and *fanjinensis* should be recognized as subspecies of *A. largeteaui*. In addition, two subspecies, *A. kuangtungensis yufeii* and *A. kuangtungensis josephi*, are described herein.

## 1. Introduction

The black-vein butterflies of the *Aporia agathon* group (concept established in this study) contains four species, including *A. agathon* (Gray, 1831) [1], *A. largeteaui* (Oberthür, 1881) [2], *A. gigantea* Koiwaya, 1993 [3] and *A. lemoulti* Bernardi, 1944 [4]. All four species are among the largest members of genus *Aporia* throughout the world. The geographical range of this group occupies a vast area from the W. Himalayas to N. Indochina and Taiwan Island—two species, *A. agathon* and *A. largeteaui*, possess the first and second widest distribution ranges, while their sibling species, *A. gigantea* and *A. lemoulti*, have a narrower range [5].

**Figure 1:**
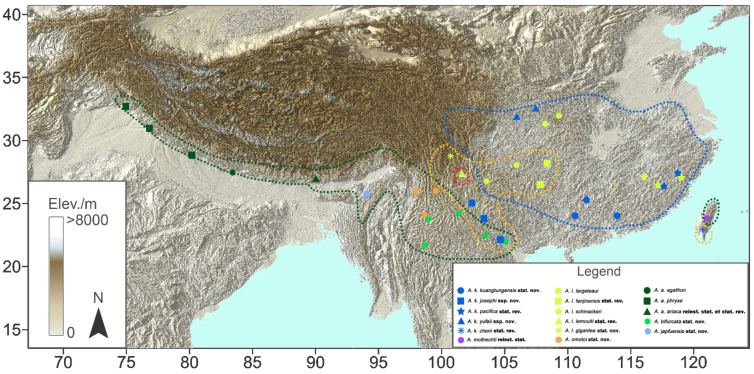
The distribution map of the species of the *Aporia agathon* group. The dotted lines indicate the distribution ranges of the four recognised species by Della Bruna et al. [5] (green: *A. agathon*; orange: *A. gigantea*; red: *A. lemoulti*; blue: *A. largeteaui*), while the points represent updated distribution localities from this study.

Within its distribution range, the wing pattern of *A. agathon* varies significantly, and thus, nine subspecies have been recognised to date, viz., ssp. *phryxe* (Boisduval, 1836) [6] from the W. Himalayas in N.W. India (Himachal Pradesh and Uttar Pradesh), ssp. *caphusa* (Moore, 1872) [7] from the W. Himalayas in N.W. India (Kashmir, Himachal Pradesh, and Uttar Pradesh), ssp. *agathon* (Gray, 1831) from the W. Himalayas in Nepal (near Kathmandu), ssp. *ariaca* (Moore, 1872) [7] from the Himalayas in Bhutan, ssp. *japfuensis* Yoshino, 2015 [8] from Nagaland in N.E. India, ssp. *gaolingonshanensis* Yoshino, 2015 [8] from Gaoligong Shan in W. Yunnan of China, ssp. *omotoi* Yoshino, 2003 [9] from the southern margin of the Hengduan Mountains in N.W. Yunnan of China, ssp. *bifurcata* Tytler, 1939 [10] from the Central Yunnan Plateau, Myanmar (Shan State), ssp. *sapaensis* Funahashi, 2003 [11] from N. Vietnam (Ha Giang), and ssp. *moltrechti* (Oberthür, 1909) from Taiwan Island of China [5,8,12] (Figure 1).

*Aporia largeteaui* exhibits high variation as well. Four named subspecies were documented inside China, with the nominotypical subspecies from S.W. to C. China (Sichuan to Hubei), ssp. *kuangtungensis* Mell, 1935 [13] from S. China (Guangxi and Guangdong), ssp. *schmackeri* (Mell, 1943) [14] from E. China (Jiangxi and Fujian), and ssp. *pacifica* (Mell, 1943) [14] also, from E. China (Zhejiang) [5]. The variation in *A. gigantea* is much simpler, but its distribution range is interesting: ssp. *gigantea* from S.W. China (Sichuan), ssp. *fanjinensis* (Yoshino, 1997) [15] from S.W. (Guizhou), and ssp. *cheni* Hsu & Chow, 1999 [16] from S.E. China (Taiwan Island) with no population in the mainland between them (Figure 1). Furthermore, [5,17] included unnamed subspecies of *A. largeteaui* and *A. gigantea* from N. Vietnam (Ha Giang and Lao Cai) in their publications, indicating cryptic taxa of these species in their southernmost range.

Are these to-date recognised species and subspecies sound? Recent molecular-based research on *Aporia* species discovered that *A. agathon* and *A. largeteaui* were not monophyletic (some subspecies are even split by other known species), indicating that some of their subspecies might be distinct [18]. To address this question, the present research collected series of samples and sequences across the distribution range of the *A. agathon* group and revisited the morphological and molecular evidence. By combining the latest evidence containing mitogenomes and DNA barcodes, as well as wing morphology and genitalic structures, a new classification of these species was established and is described herein.

## 2. Materials and Methods

### 2.1. Taxon Sampling and Identification

Fresh *Aporia* butterflies used in the present research were collected from 2019 to 2024 by netting and killed instantly by pinching the thorax. The legs of all freshly collected specimens were extracted and preserved in absolute ethanol in 1.5 mL Eppendorf micro-centrifuge tubes on the day of collection. The remainders of all specimens were stored in paper triangles and labelled alpha-numerically identically with the micro-centrifuge tubes for further taxonomical purpose. Dried specimens collected prior to 2019 were all stored at room temperature without rehydration and spreading; whole specimens or legs were provided by their collectors to the first author’s laboratory for analytical processes (Table 1).

Specimens were identified by Shao-Ji Hu by comparing morphological characters of wings using the guide book of Della Bruna et al. [5]. Morphologically similar species were identified with male genitalia against original descriptions and taxonomic literature of Koiwaya [3].

**Table 1:** The samples used in the present research, with specimen localities and accession numbers deposited in the GenBank and BOLD System; countries in the Locality column are formatted as two-letter ISO country codes with collecting sites. Asterisks denote samples with complete mitogenomes.

Taxon	Locality	Alt./m	Accession no.
*Aporia japfuensis* **stat. nov.** 2023JAP06 *	ID: Nagaland	N/A ^1^	PQ481756
*Aporia bifurcata* **stat. nov.** 2022BIF01 *	CN: Yunnan: Kunming	2000	PQ481753
*Aporia bifurcata* **stat. nov.** 2022BIF02 *	CN: Yunnan: Dali	2300	PQ481754
*Aporia bifurcata* **stat. nov.** 2015BIF01 *	VN: Lao Cai: Sapa	N/A	PQ481755
*Aporia moltrechti* **reinst. stat.** 1984MOL01 *	CN: Taiwan: Lala Shan	N/A	PQ481752
*Aporia moltrechti* **reinst. stat.** Pr1-1974	CN: Taiwan: Hualien	N/A	PQ450495
*Aporia moltrechti* **reinst. stat.**	CN: Taiwan: Nantou	N/A	AAGA-TWPUL2
*Aporia kuangtungensis cheni* **stat. rev.** Pr1-2482	CN: Taiwan: Kaohsiung	1600	PQ450496
*Aporia kuangtungensis cheni* **stat. rev.** 2024CHE01 *	CN: Taiwan: Kaohsiung	1600	PQ481750
*Aporia kuangtungensis cheni* **stat. rev.** 2024CHE02 *	CN: Taiwan: Pingtung	N/A	PQ481751
*Aporia kuangtungensis pacifica* **stat. rev.** 2023PAC01 *	CN: Zhejiang: Baishanzu	1840	PQ481749
*Aporia kuangtungensis pacifica* **stat. rev.** 2022PAC07	CN: Fujian: Wuyi Shan	2100	PQ450497
*Aporia kuangtungensis yufeii* **ssp. nov.** 2023KUD05 *	CN: Shaanxi: Fengxian	1600	PQ481747
*Aporia kuangtungensis yufeii* **ssp. nov.** 2023KUD06 *	CN: Shaanxi: Fengxian	1600	PQ481748
*Aporia kuangtungensis yufeii* **ssp. nov.** 2023KUD20	CN: Shaanxi: Huxian	N/A	PQ450499
*Aporia kuangtungensis yufeii* **ssp. nov.** 2023KUD18	CN: Gansu: Kangxian	1300	PQ450498
*Aporia kuangtungensis yufeii* **ssp. nov.** 2022KUD27	CN: Chongqing: Wuxi	N/A	PQ450500
*Aporia kuangtungensis kuangtungensis* **stat. nov.** 2014KUD12 *	CN: Hunan: Dawei Shan	N/A	PQ481743
*Aporia kuangtungensis kuangtungensis* **stat. nov.** 2016KUD01 *	CN: Guangxi: Mao’er Shan	N/A	PQ481742
*Aporia kuangtungensis kuangtungensis* **stat. nov.** 2024KUD01 *	CN: Guangdong: Shaoguan	800	PQ481741
*Aporia kuangtungensis josephi* **ssp. nov.** 2022KUD03 *	CN: Yunnan: Yiliang	2400	PQ481744
*Aporia kuangtungensis josephi* **ssp. nov.** 2023KUD01 *	CN: Funing	N/A	PQ481746
*Aporia kuangtungensis josephi* **ssp. nov.** 2015KUD01 *	VN: Ha Giang	N/A	PQ481745
*Aporia agathon ariaca* **reinst. stat. et stat. rev.**	BT: Yonphula	2500	AAGA-BTKYO1
*Aporia agathon ariaca* **reinst. stat. et stat. rev.**	BT: Tashiyangse	1550	AAGA-BTTBC1
*Aporia agathon phryxe*	CN: Tibet: Tongme	2450	AAGA-CHTNT2
*Aporia agathon phryxe*	IN: Mussoorie	N/A	KU640959
*Aporia agathon phryxe*	NP: Dolpo	2500	AAGA-NPDDL1
*Aporia agathon agathon* 2002AGT01 *	NP: Kathmandu	2300	PQ481740
*Aporia agathon agathon*	NP: Kathmandu	2250	AAGA-NPKPU3
*Aporia agathon agathon*	NP: Kathmandu	1600	AAGA-NPKVN1
*Aporia omotoi* **stat. nov.** 2022OMT03 *	CN: Yunnan: Deqen	1943	PQ481736
*Aporia omotoi* **stat. nov.** 2022OMT04 *	CN: Yunnan: Weixi	2200	PQ481737
*Aporia omotoi* **stat. nov.** 2022OMT05 *	CN: Yunnan: Zhongdian	3200	PQ481738
*Aporia omotoi* **stat. nov.** 2019OMT11	CN: Yunnan: Gongshan	1700	PQ450501
*Aporia omotoi* **stat. nov.** 2023OMT07 *	CN: Yunnan: Yingjiang	1800	PQ481739
*Aporia omotoi* **stat. nov.**	CN: Tibet: Tongme	2450	AAGA-CHTNT1
*Aporia largeteaui lemoulti* **stat. rev.** 2019LEM01 *	CN: Sichuan: Miyi	2600	PQ481732
*Aporia largeteaui lemoulti* **stat. rev.** 2019LEM02 *	CN: Sichuan: Miyi	2600	PQ481733
*Aporia largeteaui largeteaui* 2022LAG04 *	CN: Hubei: Baokang	1000	PQ481730
*Aporia largeteaui largeteaui* 2022LAG05 *	CN: Guizhou: Tongren	840	PQ481731
*Aporia largeteaui schmackeri* 2022SCM05	CN: Fujian: Wuyi Shan	1000	PQ450502
*Aporia largeteaui schmackeri* 2022SCM08	CN: Fujian: Wuyi Shan	1000	PQ450503
*Aporia largeteaui gigantea* **stat. nov.** 2017GIG07	CN: Sichuan: Ya’an	940	PQ450504
*Aporia largeteaui gigantea* **stat. nov.** 2017GIG10	CN: Sichuan: Ya’an	940	PQ450505
*Aporia largeteaui fanjinensis* **stat. rev.** 2019GIG13	CN: Chongqing: Jinfo Shan	800	PQ450506
*Aporia largeteaui fanjinensis* **stat. rev.** 2019GIG14	CN: Chongqing: Jinfo Shan	800	PQ450507

^1^ Data not available.

### 2.2. Morphological Comparisons

Specimens were spread for morphological comparison. Forewing lengths were measured using a ruler with 0.5 mm precision, and calculated into a range with mean ± s.d. when the number of specimens was ≥ 3 for a taxon. Wing markings were adopted as primary comparative morphological characters in this study; all specimens illustrated in this article were photographed with a medium grey background using a Fujifilm S9600 digital camera (Fujifilm, Japan), and processed into colour figures using Adobe Photoshop CS6 (Adobe Systems Inc., San Jose, CA, USA; licenced serial number 92298586703671763790).

The observation of male genitalia mainly followed Hu et al. [19]: the abdomen of a specimen was taken and heated in 10% NaOH solution at 70 °C for 1 h to digest the soft tissue. The sclerotized genitalic capsules were washed with water under a stereomicroscope to remove residual tissue and then transferred into 80% glycerol solution to render them transparent for 12 h. The resultant male genitalia were observed and photographed using a stereomicroscope with a digital camera mounted on it.

The descriptions of the taxa involved in this study are based on terminology proposed by Della Bruna et al. [5].

### 2.3. Mitogenome Sequencing and Assembly

Genomic DNA for mitogenome sequencing was extracted from all three legs using the Rapid Animal Genomic DNA Isolation Kit (Sangon Biotech, Shanghai, China), then the concentration was determined on a Qubit^®^ Flurometer (Thermo Fisher Scientific, Waltham, MA, USA) and stored in the refrigerator at −20 °C. The quality of resultant DNA was examined by 1% agarose gel electrophoresis under a Gel Image Analysis System (FR-980A, Furi Science & Technology Co., Ltd., Shanghai, China).

The extracted DNA was then sequenced on an Illumina NovaSeq 6000 System (Illu-mina Inc., San Diego, CA, USA). The chromatograms were analysed and translated with base calling to generate raw reads in FASTQ file. The quality of raw reads was evaluated and processed using fastp 0.36 [20] to obtain valid high-quality clean data.

Due to the high redundancy in high-throughput sequencing data, the de novo assembly strategy was used to align the reads and target sequences bidirectionally using SPAde 3.15 [21]. Sequencing errors were corrected first on the input sequences, and then the sequence was spliced using multiple Kmer values. Once the splicing was completed, BLASTn (https://blast.ncbi.nlm.nih.gov/Blast.cgi; accessed on 1 July 2022) was applied to compare scaffolds with the NCBI nucleotide library to assess similarity, and to extract the sequencing depth and coverage information of each scaffold. After filling gaps between all contigs using GapFiller 1.11 [22], the PrInSeS-G method [23] was applied to correct clipping errors and indels of small fragments during splicing. Further, built-in scripts were employed to process and evaluate contigs to obtain a complete circular genome.

In total, 25 mitogenomes were successfully sequenced and assembled. The mitogenomes of *Aporia* are circular and approximately 15–16 kb in length, containing 37 genes (13 protein coding genes (PCGs), 22 transfer RNA genes and two ribosomal RNA genes) [24,25]. tBLASTn (https://blast.ncbi.nlm.nih.gov/Blast.cgi; accessed on 1 July 2022) and GeneWise (https://www.ebi.ac.uk/jdispatcher/psa/genewise; accessed on 1 July 2022) were used to perform reverse comparison with reference genomes of *Aporia largeteaui* (MW284889), *A. bieti* (Oberthür, 1881) (KX495165) [24] and *A. hastata* (Oberthür, 1892) (OP373108) [25] to obtain PCG boundaries. The tRNA regions were annotated using MiTFi [26], and CMsearch [27] was employed with the Rfam database [28] for non-coding rRNA identification.

### 2.4. DNA Barcode Sequencing

Samples unable to produce mitochondrial genomes were sequenced for the 658 bp DNA barcode fragments. Before DNA extraction, the legs of each individual were extracted from ethanol and rehydrated with 1 mM Tris-EDTA solution (Solarbio Life Sciences, Beijing, China) in a 1.5 mL Eppendorf micro-centrifuge tube at 4 °C for 12 h to guarantee complete digestion. The rehydrated tissue was then homogenised and the genomic DNA was extracted using the TianGen TIANamp Genome DNA Kit (TianGen Biotech Co., Ltd., Beijing, China), all extraction operations followed the Kit’s instruction manual. The genomic DNA was stored at −40 °C until PCR amplification.

The PCR reaction was performed using TaKaRa Ex *Taq* Kit (TaKaRa, Dalian, China) in a 25-μL reaction system containing 2.5 μL of 10× PCR buffer, 2.0 μL of MgCl_2_ (25 mM), 2.0 μL of dNTPs mixture (2.5 mM each), 0.5 μL of each of the forward and reverse primers (20 μM), 0.25 μL of Ex *Taq* DNA polymerase (5 U/μL), and 1.0 μL of the genomic DNA template. The thermal profile for the PCR consisted of an initial denaturation at 95 °C for 3 min, followed by 30 cycles of denaturation at 94 °C for 1 min, annealing at 50 °C for 1 min, extension at 72 °C for 1 min, and finally an extension at 72 °C for 5 min. The PCR primers for DNA barcode (*cox1* gene fragment) are LCO1490 (5′- GGT CAA CAA ATC ATA AAG ATA TTG G-3′) and HCO2198 (5′-TAA ACT TCA GGG TGA CCA AAA AAT CA-3′) [29].

The quality of all PCR products was inspected using 1% agarose gel electrophoresis at 120 V for 30 min. Products with clear single bands were sequenced on an ABI 3730xl Prism automatic sequencer (Applied BioSystems, Foster City, CA, USA) by TsingKe Biotechnology Co., Ltd. (Beijing, China).

Raw sequences were aligned and proofread using the CLUSTAL W algorithm [30] in BioEdit 7.0.9 [31] and any sequence containing double peaks in the chromatograms was strictly excluded. The product sequences were checked by BLASTn against the genomic references and nucleotide collection in NCBI (https://blast.ncbi.nlm.nih.gov/; accessed on 1 July 2022). Amino acid translation was realised with the invertebrate mitochondrial criterion in MEGA X [32] to detect possible *Numts* (nuclear copies of mtDNA fragments). A search for nonsynonymous mutations, inframe stop codons, and indels was carried out to further minimise the existence of cryptic *Numts* [33,34].

### 2.5. Phylogenetic Analysis

The 25 mitogenomes (53.2%) were used as the backbone of the phylogenetic analysis, and 22 DNA barcode fragments (46.8%) were filled in to increase sample size [35]. A Bayesian Inference (BI) tree was reconstructed with the most appropriate nucleotide substitution model recovered by ModelFinder [36] in PhyloSuite 1.2.3 [37] for 10-million-generation Markov Chain Monte Carlo (MCMC) (sampled every 1000 generations with a 25% burn-in) to calculate the clade posterior probabilities (PP). The average standard deviation of split frequencies (ASDF), the potential scale reduction factor (PSRF) and the effective sample size (ESS) were adopted to evaluate the robustness of the BI analysis. Robustness was considered good when ASDF approaches 0, PSRF approaches 1, and ESS >> 200 [38,39]. An IQ-Tree was also reconstructed using the Maximum Likelihood (ML) method with the most appropriate nucleotide substitution model recovered by ModelFinder [36] in PhyloSuite 1.2.3 [37]. A bootstrap process with 10,000 iterations was performed to test the robustness of the tree topology. An individual of *Aporia melania* (Oberthür, 1892) [40] and of *A. larraldei* (Oberthür, 1876) [41] were used as the outgroup because they are the neighbouring taxa branch before the focal group in our unpublished phylogenetic research using mitochondrial genomes. The resultant tree files were annotated using FigTree 1.4.4 [42] and Adobe Illustrator CS6 (Adobe Systems Inc., San Jose, CA, USA; licenced serial number 9229-8586-7036-7176).

Phyletic properties of each recognised taxon were assessed using an online tool, Monophylizer (http://monophylizer.naturalis.nl/; accessed on 20 October 2024) [43]. Taxa identified as monophyletic were treated as good species or subspecies, while those identified as paraphyletic were further analysed using morphological characters and geographical ranges.

The Kimura two-parameter (K2P) distances [44] between identified taxa were calculated from the DNA barcode (*cox1* gene fragment) sequences by MEGA X, and visualised as a lower-triangle matrix using ‘pheatmap’ package 1.0.12 (Pretty Heatmap) [45] implemented in R 4.3.0 (https://www.r-project.org/ (accessed on 11 June 2023)).

## 3. Results

### 3.1. Phylogenetics of the Aporia agathon Group

**Figure 2:**
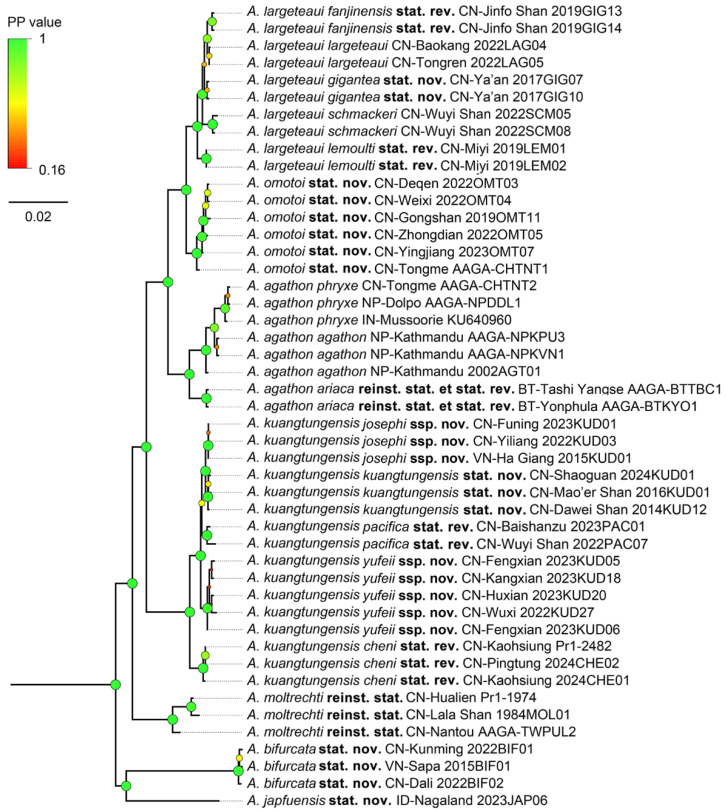
The Bayesian phylogenetic tree of all recognised taxa, with outgroups excluded. Circles at nodes indicate the posterior probability values, circle size is proportional with the posterior probability values.

**Figure 3:**
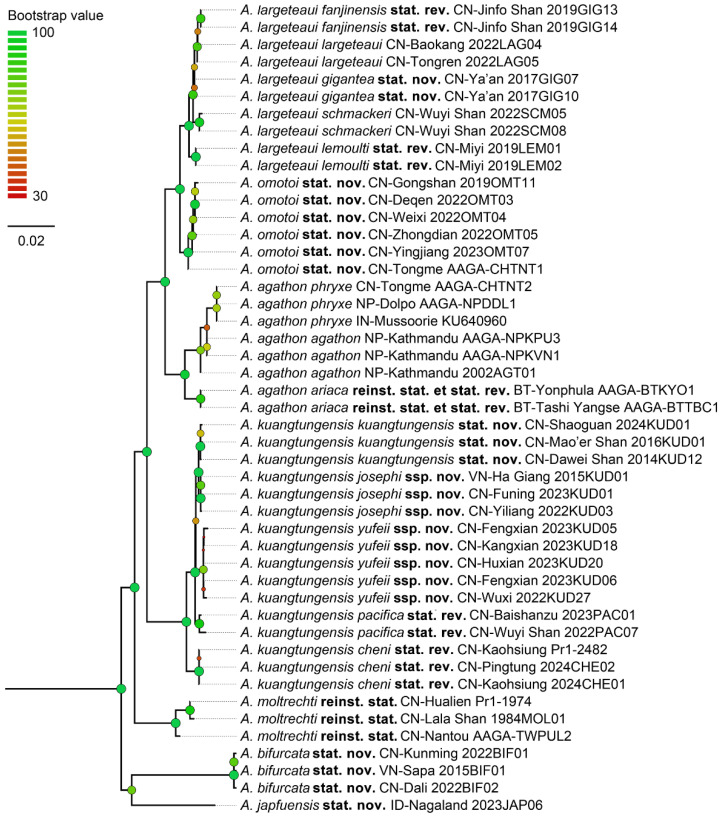
The IQ-tree of all recognised taxa, with outgroups excluded. Circles at nodes indicate the bootstrap values with 10,000 iterations, circle size is proportional with the bootstrap values.

Bayesian Inference produced a robust result with ESS = 5086.59, average PSRF = 1.000 (maximal PSRF = 1.002), and ASDF = 0.003574. Sixty-one percent of the nodes separating taxa exceed 0.90, if they are not maximal (Figure 2 and Figure S1). The IQ-Tree also produced a robust result with log-likelihood of the consensus tree reaching −35388.86, and 61% of the nodes on the BI tree being greater than 0.90, if not maximal (Figure 3 and Figure S2).

The Bayesian Inference and IQ-Tree analyses showed similar results, with only the positions of a few subspecies of *A. largeteaui* and *A. kuangtungensis* stat. nov. being different from each other. *A. japfuensis* **stat. nov.** and *A. bifurcata* **stat. nov.** are sisters to each other and basal to all other taxa. The insular species, *A. moltrechti* **reinst. stat.** from Taiwan Island, branched separately. *A. kuangtungensis* and its subspecies, *e.g*., ssp. *cheni* **stat. rev.**, ssp. *yufeii* **ssp. nov.**, ssp. *pacifica* **stat. rev.**, ssp. *kuangtungensis*, and ssp. *josephi* **ssp. nov.**, formed a large clade. From there, the three subspecies of *A. agathon* formed a clade, while *A. omotoi* **stat. nov.** split next and became a sister to another clade containing various subspecies of *A. largeteaui*, e.g., ssp. *lemoulti* **stat. rev.**, ssp. *largeteaui*, ssp. *fanjinensis* **stat. rev.**, and ssp. *gigantea* **stat. nov.** (Figure 2, Figure 3, Figures S1 and S2).

The Monophylizer analysis identified 16 taxa out of 17 as monophyletic for the Bayesian tree, with *A. agathon agathon* being detected as paraphyletic and entangled with *A. agathon phryxe*. The analysis identified 15 taxa out of 17 as monophyletic for the IQ-Tree, with *A. agathon agathon* being detected as paraphyletic and entangled with *A. agathon phryxe*, and *A. largeteaui largeteaui* also being detected as paraphyletic and entangled with *A. largeteaui fanjinensis***stat. rev.** (Table 2).

**Figure 4:**
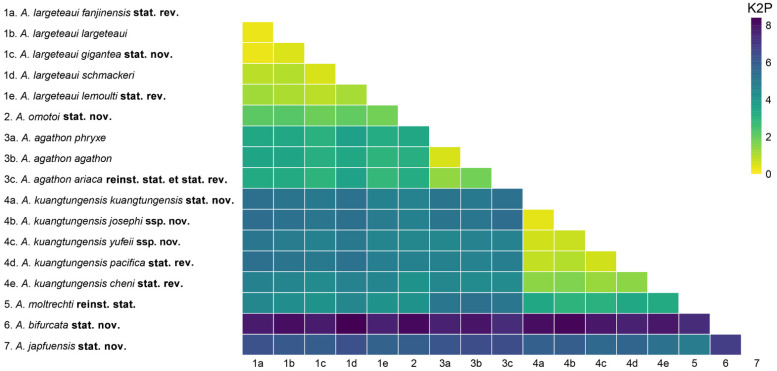
The Kimura two-parameter (K2P) genetic distances between all taxa in the present research.

The Kimura 2-parameter (K2P) genetic distance (Figure 4; Table S1) computed between *A. japfuensis* **stat. nov.** and all other taxa ranged 4.90–6.88%, between *A. bifurcata* **stat. nov.** and all other taxa (excluding *A. japfuensis* stat. nov.) ranged 7.32–8.41%. The K2P distances between *A. omotoi* **stat. nov.** and all subspecies of *A. largeteaui* ranged from 1.80 to 2.22%, while those between it and the remaining taxa were all over 3.13%. Likewise, the K2P distances between each subspecies of *A. largeteaui* and other taxa (excluding *A. omotoi* **stat. nov.**) were all over 3.02%. The K2P distances between every subspecies of *A. agathon* and *A. omotoi* **stat. nov.** were all greater than 3.13%, and those between *A. bifurcata* **stat. nov.** and *A. japfuensis* **stat. nov.** were all over 6.17%. For all subspecies of *A. kuangtungensis* stat. nov., the K2P distances between them and all subspecies of *A. largeteaui* were over 4.42%, and those between them and the remaining taxa are greater than 2.99%. It is notable that the K2P distances between two subspecies of the previous *A. gigantea* (*gigantea* and *fanjinensis*) and *A. largeteaui* (*largeteaui* and *schmackeri*) were only 0.31% and 1.00%, while that between the previous *A. lemoulti* and *A. largeteaui* was only 1.08%. The K2P distances between the five subspecies of *A. largeteaui* ranged from 0.31 to 1.23%, and those between the four subspecies of *A. kuangtungensis* **stat. nov.** ranged from 0.36 to 1.46%.

**Table 2:** The Monophylizer assessments of the phyletic properties of all recognised taxa in this study.

Taxon	Bayesian Tree		IQ-Tree	
	Assessment	Tanglees	Assessment	Tanglees
*A. largeteaui gigantea* **stat. nov.**	monophyletic	—	monophyletic	—
*A. largeteaui schmackeri*	monophyletic	—	monophyletic	—
*A. largeteaui fanjinensis* **stat. rev.**	monophyletic	—	monophyletic	—
*A. largeteaui largeteaui*	monophyletic	—	paraphyletic	*A. l. fanjinensis*
*A. largeteaui lemoulti* **stat. rev.**	monophyletic	—	monophyletic	—
*A. omotoi* **stat. nov.**	monophyletic	—	monophyletic	—
*A. agathon agathon*	paraphyletic	*A. a. phryxe*	paraphyletic	*A. a. phryxe*
*A. agathon phryxe*	monophyletic	—	monophyletic	—
*A. agathon ariaca* **reinst. stat. et stat. rev.**	monophyletic	—	monophyletic	—
*A. kuangtungensis kuangtungensis* **stat. nov.**	monophyletic	—	monophyletic	—
*A. kuangtungensis josephi* **ssp. nov.**	monophyletic	—	monophyletic	—
*A. kuangtungensis yufeii* **ssp. nov.**	monophyletic	—	monophyletic	—
*A. kuangtungensis pacifica* **stat. rev.**	monophyletic	—	monophyletic	—
*A. kuangtungensis cheni* **stat. rev.**	monophyletic	—	monophyletic	—
*A. moltrechti* **reinst. stat.**	monophyletic	—	monophyletic	—
*A. bifurcata* **stat. nov.**	monophyletic	—	monophyletic	—
*A. japfuensis* **stat. nov.**	monophyletic	—	monophyletic	—

### 3.2. Revised Species and Subspecies Checklist

#### 3.2.1. *Aporia japfuensis* Yoshino, 2015 **stat. nov.**

*Aporia agathon japfuensis* Yoshino, 2015; Butterfly Science, 2: 25, f. 1–4; TL: “Jafpu, alt, 1800 m, Nagaland, N.E. India” [8].

**Figure 5:**
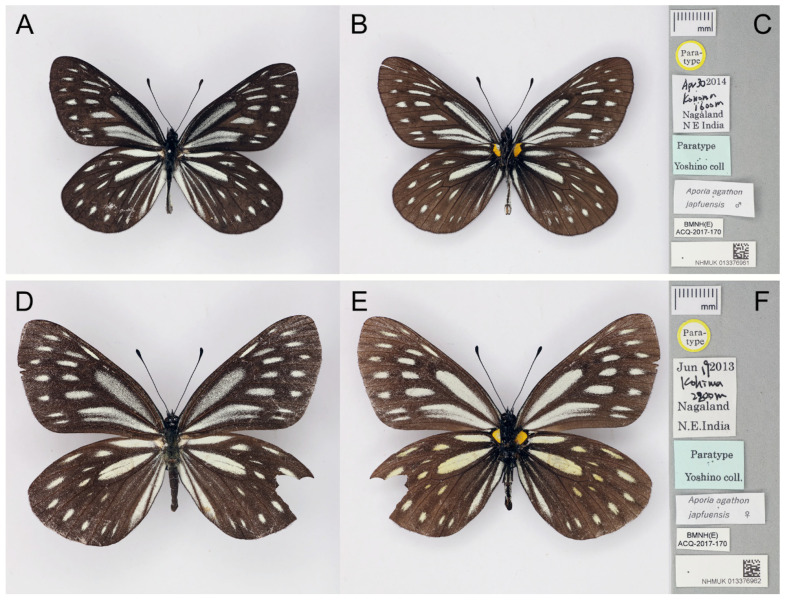
The paratypes of *Aporia agathon japfuensis* Yoshino, 2015 deposited in the Natural History Museum, London, UK. (**A**–**C**): male; (**A**): upperside; (**B**): underside; (**C**): labels: round-type label with yellow circle and printed “Para-type”, the first rectangular label with both printed and K. Yoshino’s handwritten “Apr 30 2014/Konoma/1600m/Nagaland/N E India”, the second (light blue) rectangular label with printed “Paratype/Yoshino coll.”; the third rectangular label with printed “*Aporia agathon/japfuensis* ♂.”; the fourth rectangular label with printed “BMNH(E) ACQ-2017-170”; the last (bottom) rectangular label bears an accession number and a QR code of the NHM; scale bar = 10 mm. (**D**–**F**): female; (**D**): upperside; (**E**): underside; (**F**): labels: round-type label with yellow circle and printed “Para-type”, the first rectangular label with both printed and K. Yoshino’s handwritten “Jun 19 2013/Kohima/2800m/Nagaland/N E India”, the second (light blue) rectangular label with printed “Paratype/Yoshino coll.”; the third rectangular label with printed “*Aporia agathon/japfuensis* ♀.”; the fourth rectangular label with printed “BMNH(E) ACQ-2017-170”; the last (bottom) rectangular label bears an accession number and a QR code of the NHM; scale bar in mm. Type specimen photographs © Copyright Trustees of the Natural History Museum, used with permission under Creative Commons License 4.0 (https://creativecommons.org/licenses/by/4.0/). Any other use of this image, except for personal study, requires prior written consent of the housing institution.

**Diagnostic characters (Figure 5):** Forewing length: male 40 mm, female 43 mm (both measured from type photos). Both wings slightly elongate. Upperside blackish with narrow and small ivory-coloured markings, forewing markings usually dusted with blackish scales, while hindwing ones often clean, discocells with or without black lines. Underside blackish brown, markings ivory-coloured and less dusted with blackish scales, yellow hue may be present on the hindwing. Female similar to male, ground colour paler, hindwing underside markings more yellowish.

**Figure 6:**
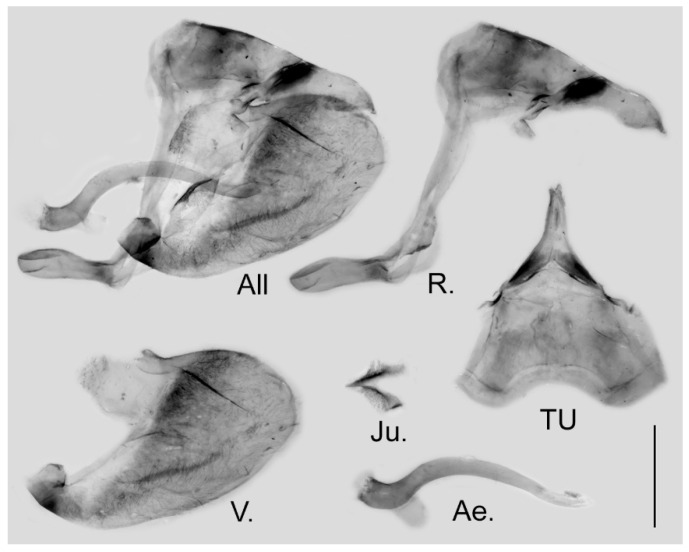
Male genitalia of *Aporia japfuensis* Yoshino, 2015 **stat. nov.** from Nagaland, India; All: entire male genitalia with left valve removed; R.: ring; TU: dorsal view of tegumen and uncus; V.: right valve; Ae.: aedeagus; Ju.: juxta; scale bar = 1 mm.

**Male genitalia (Figure 6):** Heavily sclerotized. Ring slender, anteriorly convex near tegumen, angle with saccus slightly obtuse; uncus dorsally broad at base, gradually narrowed into a bifid tip; uncus laterally slender and beak-like, dorsal margin slightly concave at basal third and convex in the remaining two-thirds, tip bifid, acute, but not evidently hooked; saccus of moderate length with expanded tip. Valve elongate, heart-shaped, both dorsal and ventral margins smooth, tip rounded, fovea small, ventral margin of valve below fovea thickened. Aedeagus slender, curved with a trochanter at its ventral base. Juxta V-shaped with two broad arms separated from base.

**Female genitalia:** Not examined due to lack of available material.

**Host plants:** Unknown, not stated in original description, but presumably species of family Berberidaceae.

**Distribution:** Nagaland, India [8].

#### 3.2.2. *Aporia bifurcata* Tytler, 1939 **stat. nov.**

**Figure 7:**
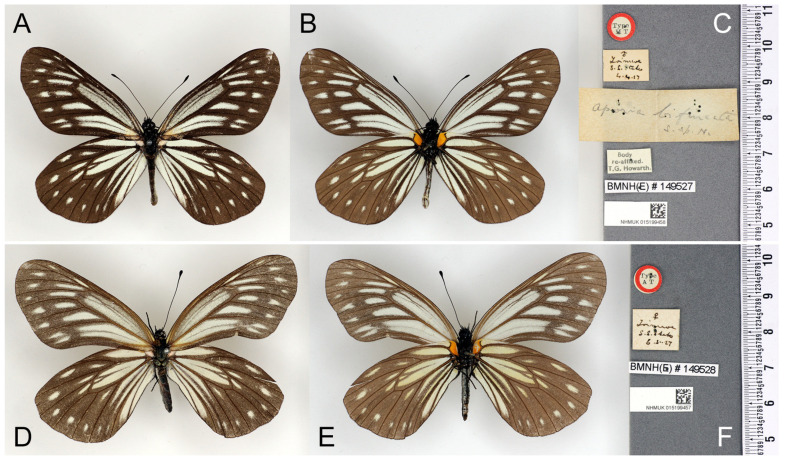
The types of *Aporia agathon bifurcata* Tytler, 1939 deposited in the Natural History Museum, London, UK; (**A**–**C**): male; (**A**): upperside; (**B**): underside, (**C**): labels: round-type label with red circle and printed “Type/HT”, the first rectangular label with Tytler’s handwritten “♂/Loimwe/S. S.[han] States/4.4.27”; the second (largest) rectangular label with Tytler’s handwritten “Aporia bifurcata/s. sp. n.”; the third rectangular label with printed ”Body/re-affixed./T.G. Howarth.”; narrow rectangular label with printed “BMNH(E) # 149527”; the last (bottom) rectangular label bears an accession number and a QR code of the NHM; scale bar in mm. (**D**–**F**): female; (**D**): upperside; (**E**): underside; (**F**): labels: round-type label with red circle and printed “Type/AT”, first rectangular label with Tytler’s handwritten “♀/Loimwe/S. S.[han] States/6.5.27”; narrow rectangular label with printed “BMNH(E) # 149528”; last (bottom) rectangular label bears an accession number and a QR code of the NHM; scale bar in mm. Type specimen photographs © Copyright Trustees of the Natural History Museum, used with permission under Creative Commons License 4.0 (https://creativecommons.org/licenses/by/4.0/). Any other use of this image, except for personal study, requires prior written consent of the housing institution.

*Aporia agathon bifurcata* Tytler, 1939; J. Bombay nat. Hist. Soc., 41: 240; TL: “Loimwe, S Shan State” [Myanmar] (Figure 7) [10].
  *Aporia agathon sapaensis* Funahashi, 2003; Wallace, 8: 6; TL: Sapa, Lao Cai, N. Vietnam [11].

**Figure 8:**
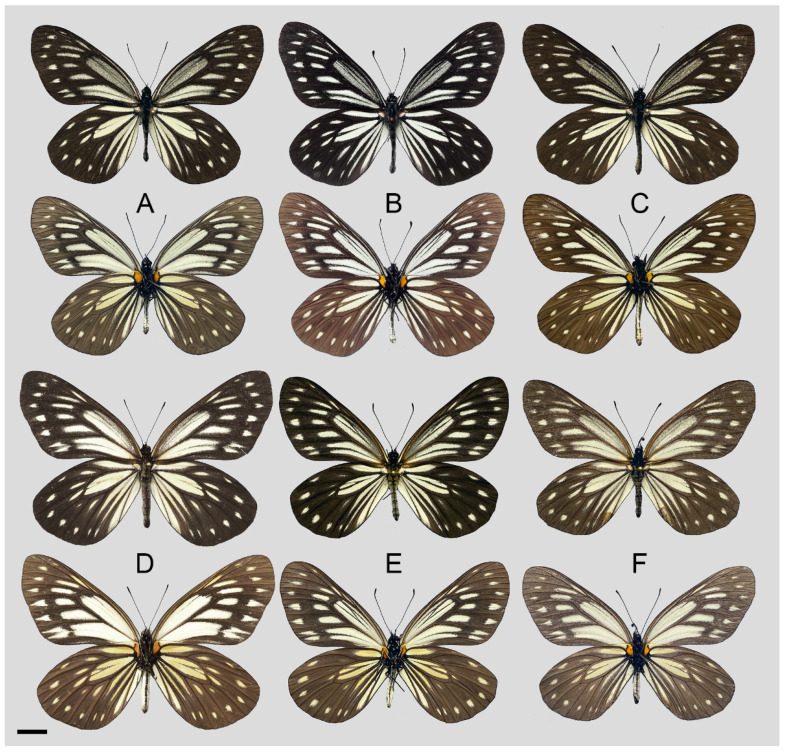
*Aporia bifurcata* Tytler, 1939 **stat. nov.**, (**A**–**C**) male, (**D**–**F**) female; (**A**): Yangbi, Yunnan, China; (**B**): Xundian, Yunnan, China; (**C**): Luliang, Yunnan, China; (**D**): Dali, Yunnan, China; (**E**): Kunming, Yunnan, China; (**F**): Xinping, Yunnan, China; upperside above, underside below, scale bar = 10 mm.

**Diagnostic characters (Figure 8):** Forewing length: male 38–42 mm (mean = 40.9 ± 2.6 mm, *n* = 51), female 42–48 mm (mean = 45.4 ± 2.4 mm, *n* = 5). Both wings narrow and elongate. Upperside blackish with ivory-coloured markings, discocells with bifid black lines, discocell and postdiscal markings on forewing upperside heavily irrigated with dark scales. Underside brown, markings ivory-coloured, a light-yellow hue always presents on the hindwing. Female similar to male, ground colour paler, hindwing markings yellowish.

**Figure 9:**
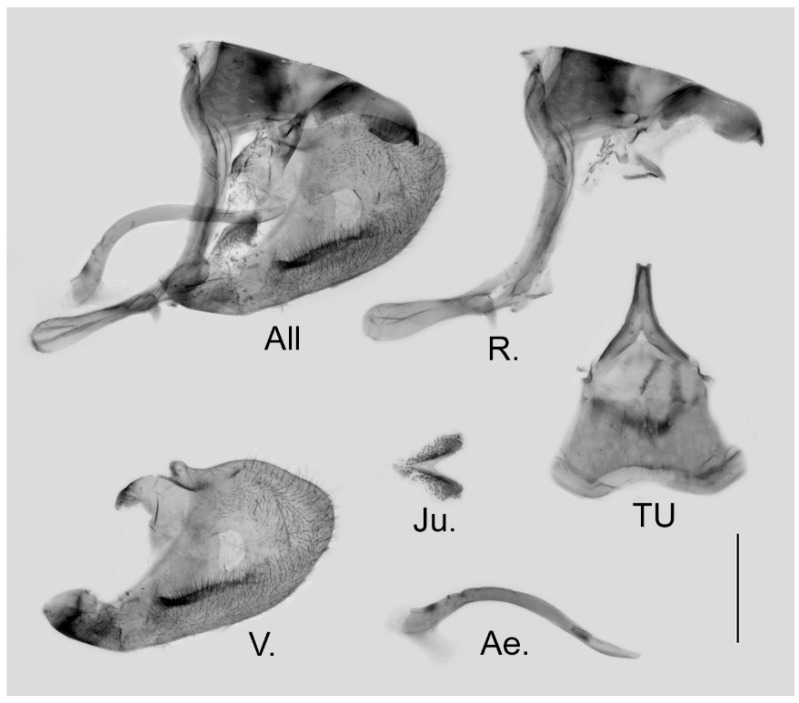
Male genitalia of *Aporia bifurcata* Tytler, 1939 **stat. nov.** from Kunming, Yunnan, China; All: entire male genitalia with left valve removed; R.: ring; TU: dorsal view of tegumen and uncus; V.: right valve; Ae.: aedeagus; Ju.: juxta; scale bar = 1 mm.

**Male genitalia (Figure 9):** Heavily sclerotized. Ring slender, anteriorly curved near tegumen, angle with saccus obtuse; uncus dorsally broad at base, gradually narrowed into a small parallel bifid tip; uncus laterally beak-like, dorsal margin slightly concave at basal third, and smoothly curved ventrally in the remaining two-thirds, tip bifid and hooked; saccus long and narrow. Valve elongate, approximately heart-shaped, both dorsal and ventral margins smooth, tip rounded, fovea large and oval, ventral margin of valve below fovea thickened. Aedeagus slender, curved with a trochanter at its ventral base. Juxta broad V-shaped with two arms separated from base.

**Figure 10:**
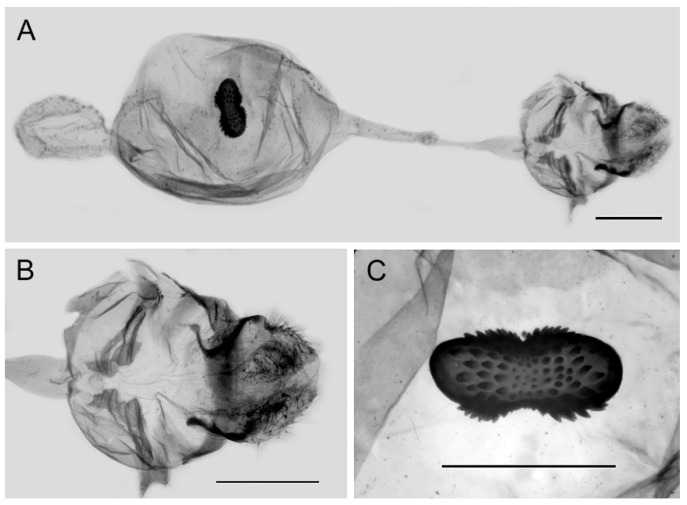
Female genitalia of *Aporia bifurcata* Tytler, 1939 **stat. nov.** from Kunming, Yunnan, China; (**A**): entire female genitalia; (**B**): enlarged ostium and sterigma; (**C**): enlarged signum; scale bars = 1 mm.

**Female genitalia (Figure 10):** Papillae anales elongate, tip rounded, covered with dense setae. Apophyses posteriors in long rod shape, apophyses anteriores in short conical shape. Lamella antevaginalis connects a pair of hairy double-folded sterigma, with the basal part shorter triangular and the distal part slender finger-shaped, lamella postvaginalis slightly sclerotized. Ductus bursae tubular and membranous, rather slender, but broader near base. Corpus bursae oval with appendix bursae attached anteriorly, signum bowknot-shaped, mostly covered with coarse spines (spines in the central part finer) except for two tips.

**Host plants:** *Berberis pruinosa* (Berberidaceae) (field observation by the first author).

**Distribution:** Myanmar: Shan State [10]; China: W., C., E., and S.E. Yunnan; Vietnam: Lao Cai and Ha Giang [46].

**Notes:** Funahashi [11] described *Aporia agathon sapaensis* based on elongate wing shape and paler colouration of specimens collected from Sapa, Lao Cai Province of N. Vietnam. However, those characters are also common in populations in Yunnan, which could only be individual variations without geographic association. Yoshino [8] treated *sapaensis* as a synonym of *bifurcata*, and the present study agrees with this treatment.

#### 3.2.3. *Aporia moltrechti* (Oberthür, 1909) **reinst. stat.**

*Pieris Moltrechti* Oberthür, 1909; Bull. Soc. ent. Fr., 1909: 48; TL: “Central Formose; Arrizan;, vis à vis le Mont-Morrison; District Kaji” [Ali Shan, C. Taiwan, China] [47].
  *Delias taiwana* Wileman, 1909; Annot. zool. Japon., 7 (2): 95; TL: “Formosa, Arisan” [Ali Shan, Taiwan, China] [48].
  *Aporia agathon moltrechti* (Oberthür, 1909) (Fruhstorfer [49]).

**Figure 11:**
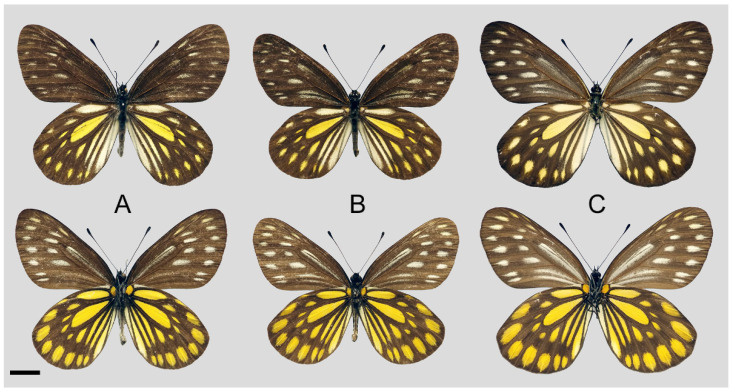
*Aporia moltrechti* (Oberthür, 1909) **reinst. stat.**, (**A**,**B**) male, C female; (**A**–**C**): Lala Shan, Taiwan, China; upperside above, underside below, scale bar = 10 mm.

**Diagnostic characters (Figure 11):** Forewing length: male 35–40 mm (mean = 36.8 ± 2.2 mm, *n* = 5), female 38–43 mm (mean = 39.3 ± 2.2 mm, *n* = 5). Both wings broad and rounded. Upperside forewing blackish with rather reduced and obscure greyish to whitish markings in postdiscal to subterminal areas, discocells usually without whitish lines; hindwing blackish with yellow discocell, postdiscal, subterminal markings, and cream humeral and tornal markings; blackish fine line in discocell. Underside forewing brownish black, whitish markings more evident than on upperside, hindwing bright yellow with thick black veins and postdiscal band. Female similar to male, ground colour paler, whitish and yellow markings larger and evident.

**Figure 12:**
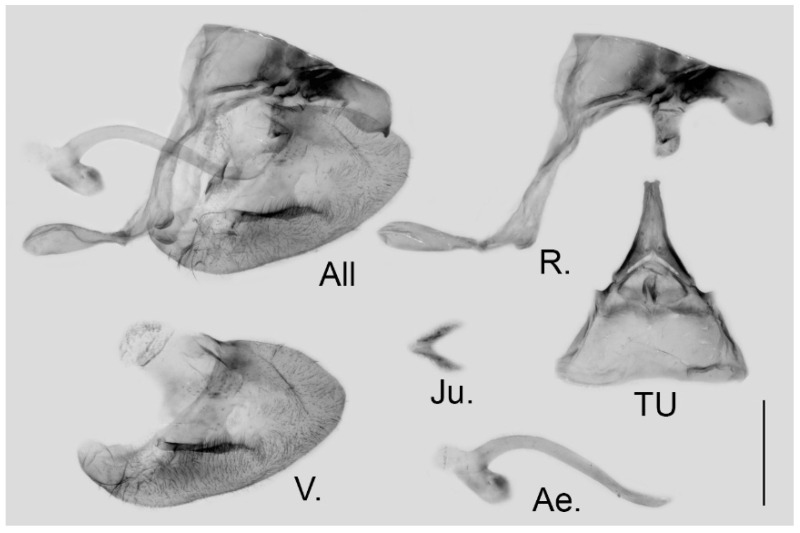
Male genitalia of *Aporia moltrechti* (Oberthür, 1909) **reinst. stat.** from Taiwan Island, China; All: entire male genitalia with left valve removed; R.: ring; TU: dorsal view of tegumen and uncus; V.: right valve; Ae.: aedeagus; Ju.: juxta; scale bar = 1 mm.

**Male genitalia (Figure 12):** Heavily sclerotized. Ring slender, anteriorly curved near tegumen, angle with saccus slightly obtuse; uncus dorsally broad at base, gradually narrowed into a shallowly bifid tip; uncus laterally beak-like, dorsal margin concave at basal third and convex in the remaining two-thirds, tip bifid and hooked; saccus in moderate length with expanded tip. Valve evidently elongate, approximately heart-shaped, both dorsal and ventral margins smooth, tip rounded, fovea oval, ventral margin of valve below fovea thickened. Aedeagus slender, curved with a trochanter at its ventral base. Juxta slender V-shaped with two arms separated from base.

**Figure 13:**
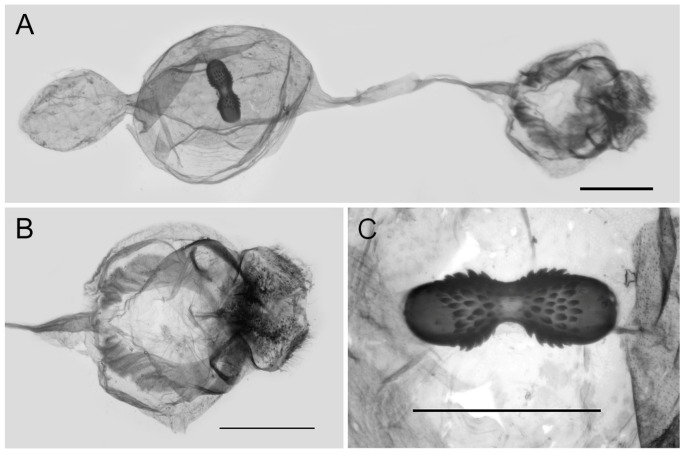
Female genitalia of *Aporia moltrechti* (Oberthür, 1909) **reinst. stat.** from Taiwan Island, China; (**A**): entire female genitalia, (**B**): enlarged ostium and sterigma, (**C**): enlarged signum; scale bars = 1 mm.

**Female genitalia (Figure 13):** Papillae anales elongate, tip rounded, covered with dense setae. Apophyses posteriors in long rod shape, apophyses anteriores in short conical shape. Lamella antevaginalis connects a pair of hairy, closely connected sterigma, with the basal and distal parts of equal lengths and similar shape, lamella postvaginalis slightly sclerotized. Ductus bursae tubular and membranous, rather slender, but broader near base. Corpus bursae oval with appendix bursae attached anteriorly, signum bowknot-shaped, only base of expanded part covered with coarse spines.

**Host plants:** *Berberis kawakamii*, *B. brevisepta*, *Mahonia oiwakensis* (Berberidaceae) [12].

**Distribution:** Endemic to Taiwan Island of China.

#### 3.2.4. *Aporia kuangtungensis* Mell, 1935 **stat. nov.**

*Aporia largeteaui kuangtungensis* Mell, 1935; Mitt. Deut. ent. Ges., 6: 37; TL: Nordkwangtung, Mantsishan [Wanshi Shan, Renhua, N. Guangdong, China] [13].

**Distribution and subspecies:** China: Shaanxi, N. Sichuan, N. Chongqing, Yunnan, S. Hunan, N. Guangxi, N. Guangdong, Fujian, Zhejiang, and Taiwan; Vietnam: Lao Cai and Ha Giang. Three subspecies are recognised from the west to the east in its range.

**Figure 14:**
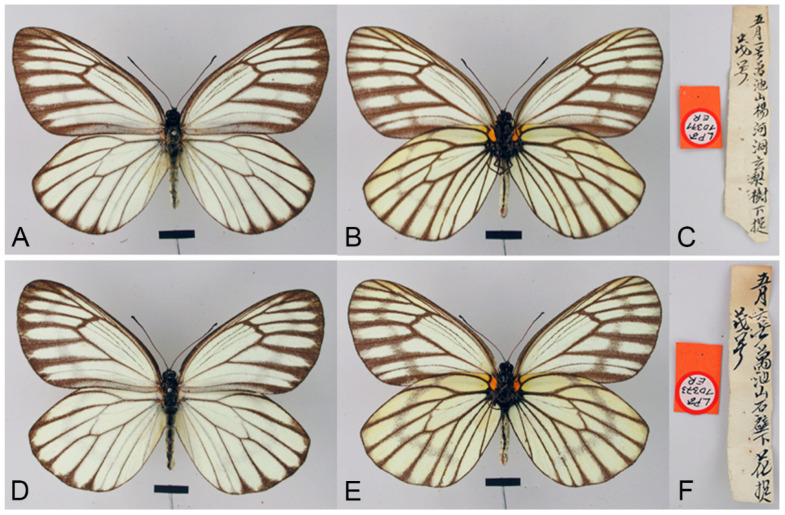
The types of *Aporia largeteaui kuangtungensis* Mell, 1935 (males) deposited in the Museum für Naturkunde der Humboldt-Universität, Berlin, Germany; (**A**,**D**): upperside; (**B**,**E**): underside; (**C**,**F**): labels: (**C**): round-type label with red circle and “LP♂/10371/ER” written; the long rectangular label with black Chinese handwriting translated “Collected under the *xuanli* tree at Yanghe Cave, Wanchi Shan [‘Mantsishan’ is the Cantonese pronunciation], May 1st [year not noted]”; (**F**): round-type label with red circle and written “LP♂/10373/ER”, the long rectangular label with black Chinese handwriting translated “Collected on flowers near a stone cliff of Wanchi Shan, May 6th [year not noted]”; scale bar = 10 mm. Type specimen photographs © Copyright Museum für Naturkunde der Humboldt-Universität, used with permission under Creative Commons License 4.0 (https://creativecommons.org/licenses/by/4.0/). Any other use of this image, except for personal study, requires prior written consent of the housing institution.

**Note:** The type locality “Mantsishan” documented in the literature originated from the Cantonese pronunciation of Wanshi Shan, a mountain in Renhua County of northern Guangdong Province, China (Figure 14).

##### *Aporia kuangtungensis yufeii* Hu & Zhang **ssp. nov.**

urn:lsid:zoobank.org:act:C918ABB8-3213-4867-8CE1-F682572274D8

**Figure 15:**
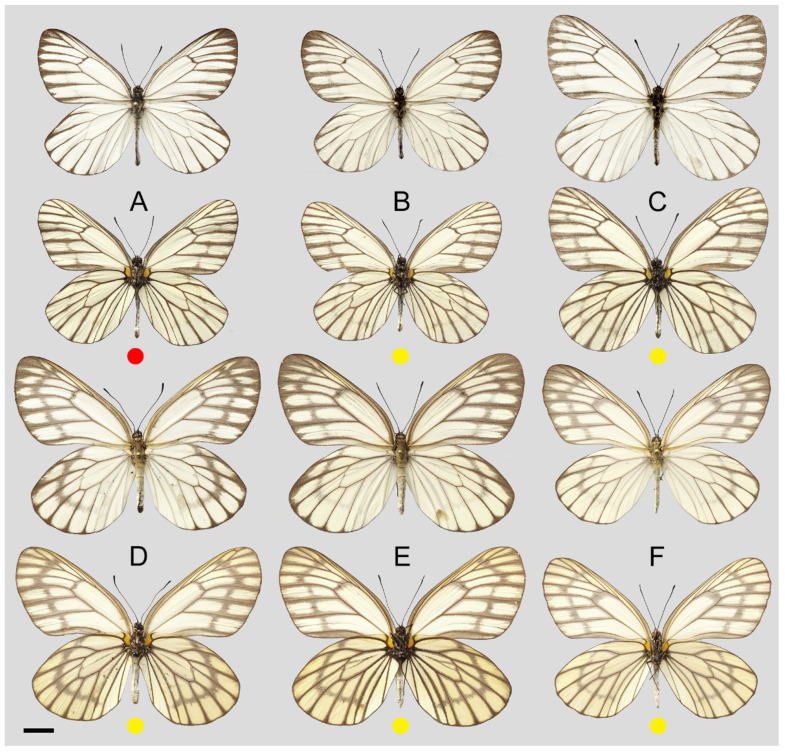
*Aporia kuangtungensis yufeii* Hu & Zhang **ssp. nov.**; (**A**–**C**) male, (**D**–**F**) female; (**A**): Ningshan, Shaanxi, China; (**B**): Huxian, Shaanxi, China; (**C**): Kangxian, Gansu, China; (**D**,**E**): Huxian, Shaanxi, China; (**F**): Zhen’an, Shaanxi, China; upperside above, underside below; red circle: HOLOTYPE; yellow circles: PARATYPES, scale bar = 10 mm.

**Description: Male (Figure 15A–C):** Forewing length 38–46 mm (mean = 42.4 ± 3.0 mm, *n* = 9). Forewing upperside cream white, costa greyish, discocell without black lines, end of discocell with short blackish band, veins blackish, thickened towards tip only from postdiscal area, termen blackish, postdiscal invisible; forewing underside similar to upperside; apical area with pale yellow hue, dorsum blackish, postdiscal band only faintly indicated. Hindwing upperside cream white, veins blackish, only thickened near termen, termen blackish; hindwing underside similar to upperside with pale yellow hue, bifid black lines faintly marked in discocell, postdiscal band only faintly indicated.

**Female (Figure 15D–F):** Forewing length 38–51 mm (mean = 46.8 ± 4.3 mm, *n* = 9). Forewing upperside cream white peppered with grey scales, costa brown, dark markings greyish black, thicker than those in male, postdiscal band better defined; forewing underside cream white, apical area with light yellow hue, dark markings as upperside. Hindwing upperside cream white, dark markings greyish black, thicker than those in male, postdiscal band better defined; hindwing underside cream yellowish white, dark markings as on upperside.

**Figure 16:**
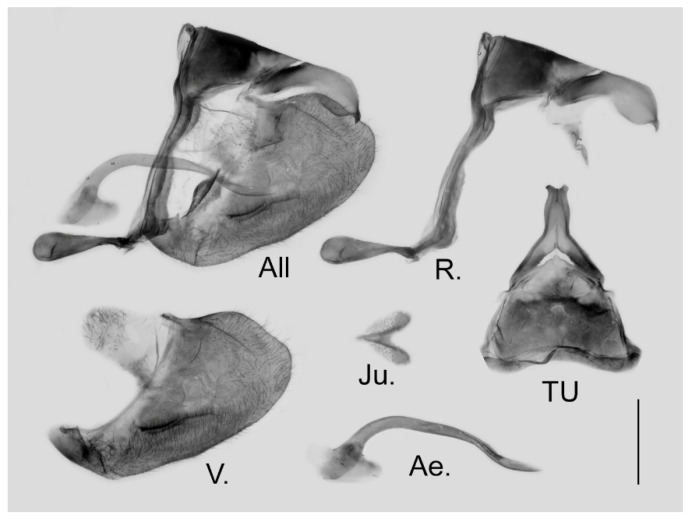
Male genitalia of *Aporia kuangtungensis yufeii* Hu & Zhang **ssp. nov.** from Ningshan, Shaanxi, China; All: entire male genitalia with left valve removed; R.: ring; TU: dorsal view of tegumen and uncus; V.: right valve; Ae.: aedeagus; Ju.: juxta; scale bar = 1 mm.

**Male genitalia (Figure 16):** Heavily sclerotized. Ring slender, straight, perpendicular with tegumen and obtuse angle with saccus; uncus dorsally broad at base, immediately narrowed into a shaft with swollen median section and outwardly bifid tip; uncus laterally beak-like, dorsal margin straight at basal two-thirds and abruptly curved ventrally at distal third, tip bifid and hooked; saccus long, narrow at base and broader on tip. Valve elongate, approximately heart-shaped, both dorsal and ventral margins smooth, tip rounded, fovea large and dorsoventrally elongate, ventral margin of valve below fovea thickened. Aedeagus slender, curved with trochanter at its ventral base. Juxta broad, V-shaped with two arms widely apart. 

**Figure 17:**
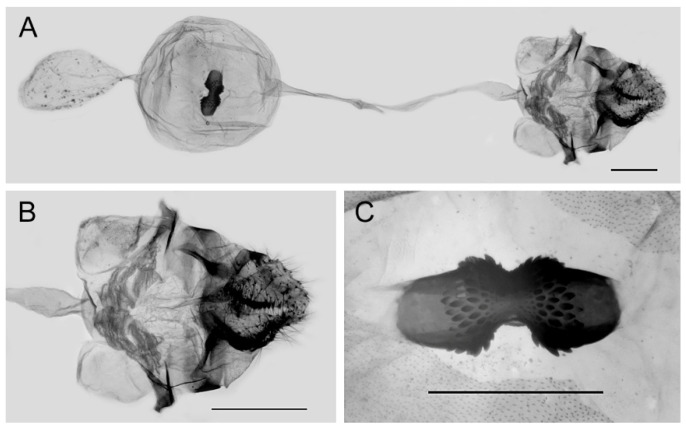
Female genitalia of *Aporia kuangtungensis yufeii* Hu & Zhang **ssp. nov.** from Huxian, Shaanxi, China; (**A**): entire female genitalia, (**B**): enlarged ostium and sterigma, (**C**): enlarged signum; scale bars = 10 mm.

**Female genitalia (Figure 17):** Papillae anales round and short, covered with dense setae. Apophyses posteriors in long rod shape, apophyses anteriores in short conical shape. Lamella antevaginalis connects a pair of hairy double-folded sterigma of equal size and nearly parallel to each other; lamella postvaginalis slightly sclerotized. Ductus bursae tubular and membranous, rather slender, but slightly broader near base. Corpus bursae oval with appendix bursae attached anteriorly, signum large, both ends expanded and rounded, bases covered with spines, median part narrower and smooth without spines.

**Differential diagnosis:** This subspecies is evidently paler than the nominotypical subspecies, especially the forewing upperside. On both wings of the male, the postdiscal bands are much reduced, and could be invisible from the upperside and only indicated on the underside. The veins on both wings are less thickened with dark scales, only evident near the termens. The female is also paler, with ground colour of cream white instead of greyish.

**Type material: HOLOTYPE:** CHINA: ♂, Guanghuojie, Ningshan, Shaanxi, 2011-VI-25, Y. F. Li *leg.* [KIZ]. **PARATYPES:** CHINA: 1♀, Donglao He, Huxian, 2010-VII-6, Y. F. Li *leg.* [KIZ]; 1♂, 1♀, Xilao He, Huxian, Shaanxi, 2011-VI-12, Y. F. Li *leg.* [KIZ]; 1♀, Nanlao He, Huxian, Shaanxi, 2011-VI-12, Y. F. Li *leg.* [KIZ]; 1♂, Huangjiawan (700 m), Zhen’an, Shaanxi, 2017-V-10 (emerge), Y. F. Li *leg.* [SJH]; 1♀, ditto, 2017-V-5 (emerge), Y. F. Li *leg.* [SJH]; 1♂, Ertiao Gou (700–1000 m), Zhen’an, Shaanxi, 2015-V-24, Y. F. Li *leg.* [SJH]; 1♀, ditto, 2014-VI-7, Y. F. Li *leg.* [SJH]; 1♂, Zhanghe, Langao, Shaanxi, 2013-VI-11, Y. F. Li *leg.* [SJH]; 1♂, Xiaonanxi (900–1000 m), Nanzhen, Shaanxi, 2012-VI-9, Y. F. Li *leg.* [SJH]; 1♀, Baiyu, Nanzhen, Shaanxi, 2017-VI-25, Y. F. Li *leg.* [SJH]; 1♂, Yunwu Shan, Shiquan, Shaanxi, 2012-VI-22, Y. F. Li *leg.* [SJH]; Qinghe (1300–1400 m), Kangxian, Gansu, 2016-VI-13, Y. F. Li *leg.* [SJH]; 1♂, 2♀♀, Wuxi, Chongqing, 2022-VI-24, Z. H. Jiang *leg.* [ZHJ]. The holotype and four paratypes were deposited in Kunming Institute of Zoology (KIZ), Chinese Academy of Sciences under the accession numbers KIZ0139646–KIZ0139650. The remaining paratypes in Shao-Ji Hu’s private collection will be transferred to the main collection in Yunnan University in the future.

**Host plants:** *Berberis soulieana* and *Mahonia fortunei* (Berberidaceae) (Y. F. Li, pers. comm.).

**Distribution:** Currently only known in the Qinling Mountains in Shaanxi and the Daba Mountains in Sichuan and Chongqing of China.

**Phenology:** Univoltine; adults fly in June.

**Etymology:** The subspecific name of this new taxon was dedicated to Prof. Yu-Fei Li, an experienced lepidopterist who studies the butterfly and moth fauna of the Qinling Mountains in Shaanxi Province, China.

##### *Aporia kuangtungensis josephi* Hu & Zhang **ssp. nov.**

urn:lsid:zoobank.org:act:A94B0DC6-9B35-4A3D-84C6-40C3A6B9682D

**Figure 18:**
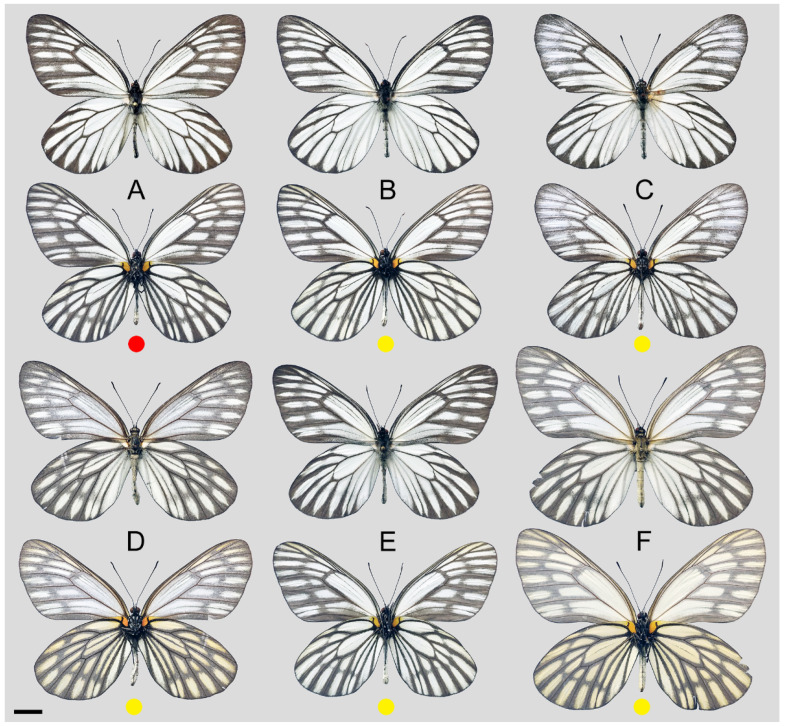
*Aporia kuangtungensis josephi* Hu & Zhang **ssp. nov.**, (**A**–**C**,**E**) male, (**D**,**F**) female; (**A**): Yiliang, Yunnan, China; (**B**,**D**): Huize, Yunnan, China; (**E**,**F**): Dongchuan, Yunnan, China; upperside above, underside below; red circle: HOLOTYPE; yellow circles: PARATYPES; scale bar = 10 mm.

**Description: Male (Figure 18A–C):** Forewing length 40–45 mm (mean = 43.0 ± 1.6 mm, *n* = 9). Forewing upperside cream white, costa greyish, two to three fine black lines in discocell, veins blackish, thickened towards tip and reduced near base, termen blackish, postdiscal band greyish, faintly marked; forewing underside similar to upperside, apical area with light yellow hue, dorsum blackish, postdiscal band more obvious. Hindwing upperside cream white, veins blackish, thickened towards tip and reduced near base; termen blackish; hindwing underside similar to upperside with light yellow hue, bifid fine black lines in discocell, postdiscal band more obvious.

**Female (Figure 18D–F):** Forewing length 46–52 mm (mean = 47.8 ± 2.2 mm, *n* = 9). Forewing upperside greyish white peppered with grey scales, costa brown, dark markings greyish black, thicker than those in male, postdiscal band better developed; forewing underside greyish white with yellow apical area, dark markings as on upperside. Hindwing upperside greyish white, dark markings greyish black, thicker than those in male, postdiscal band better developed; hindwing underside cream yellow, dark markings as on upperside, postdiscal band clearer.

**Figure 19:**
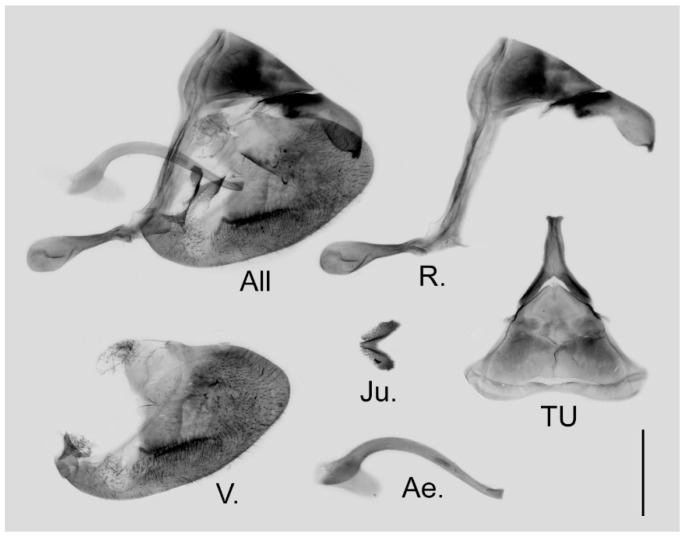
Male genitalia of *Aporia kuangtungensis josephi* **ssp. nov.** from Yiliang, Kunming, Yunnan, China; All: entire male genitalia with left valve removed; R.: ring; TU: dorsal view of tegumen and uncus; V.: right valve; Ae.: aedeagus; Ju.: juxta; scale bar = 1 mm.

**Male genitalia (Figure 19):** Heavily sclerotized. Ring slender, straight, perpendicular with tegumen and obtuse angle with saccus; uncus dorsally broad at base, immediately narrowed into a slender shaft with outwardly bifid tip; uncus laterally beak-like, dorsal margin straight at basal two-thirds and abruptly curved ventrally at distal third, tip bifid and hooked; saccus long, narrow at base and broader on tip. Valve elongate, approximately heart-shaped, both dorsal and ventral margins smooth, tip rounded, fovea large and dorsoventrally elongate, ventral margin of valve below fovea thickened. Aedeagus slender, curved with a trochanter at its ventral base. Juxta broad V-shaped with two arms widely apart.

**Figure 20:**
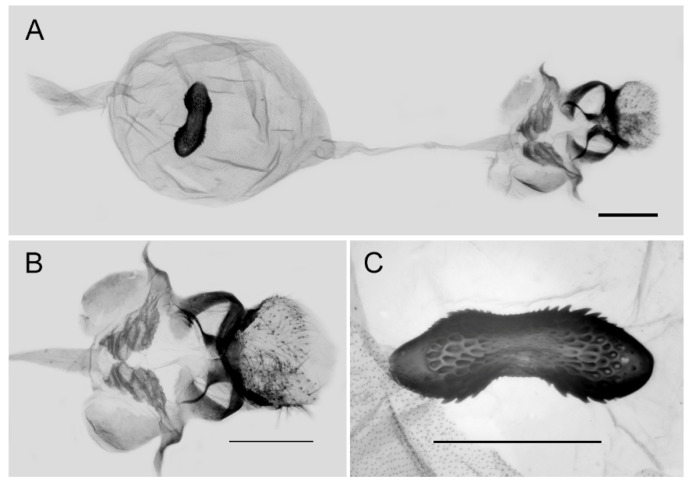
Female genitalia of *Aporia kuangtungensis josephi* **ssp. nov.** from Huize, Qujing, Yunnan, China; (**A**): entire female genitalia; (**B**): enlarged ostium and sterigma; (**C**): enlarged signum; scale bars = 1 mm.

**Female genitalia (Figure 20):** Papillae anales round and short, covered with dense setae. Apophyses posteriors in long rod shape, apophyses anteriores in short conical shape. Lamella antevaginalis connects a pair of hairy double-folded sterigma of equal size and near parallel to each other, lamella postvaginalis slightly sclerotized. Ductus bursae tubular and membranous, rather slender, but slightly broader near base. Corpus bursae oval with appendix bursae attached anteriorly, signum large, both ends expanded and rounded, covered with fine spines, median part narrower and smooth without spines.

**Differential diagnosis:** Wing morphology intermediate between *A. largeteaui* and *A. gigantea*, with black markings evidently reduced compared to *A. gigantea*, but heavier than those in typical *A. largeteaui*. The shape of uncus of male genitalia is a better distinguishing character for this species.

**Type material: HOLOTYPE:** CHINA: ♂, Goujie (2400 m), Yiliang, Kunming, Yunnan, 2018-V-10, H. H. Zhang *leg.* [KIZ]. **PARATYPES:** CHINA: 1♂, Yanshan, Wenshan, Yunnan, 2003-V-14, X. C. Liang *leg.* [KIZ]; 1♂, Baga, Yanshan, Wenshan, Yunnan, 2003-V-15, X. C. Liang *leg.* [KIZ]; 1♀, Shuangbai, Chuxiong, Yunnan, 2003-V-31, X. Y. Li *leg.* [KIZ]; 1♂, 1♀, Fazhe, Dongchuan, Kunming, Yunnan, 2014-V-2, H. H. Zhang *leg.* [SJH]; 1♂, Dongchuan, Kunming, Yunnan, 2014-V-7, local catcher *leg.* [SJH]; 4♂♂, 1♀, Leye (1810 m), Huize, Qujing, Yunnan, 2017-VI-11, S. J. Hu *leg.* [SJH]; 1♀, Keyi (2100 m), Mile, Honghe, Yunnan, 2022-VI-15, H. H. Zhang *leg.* [SJH]; 7♀♀, Goujie (2400 m), Yiliang, Kunming, Yunnan, 2022-VI-17, S. J. Hu *leg.* [SJH]. 1♂, Xiaonanjia, Funing, Yunnan, 2023-IV-22, H. H. Zhang *leg.* [SJH]. VIETNAM: 1♂, Ha Giang, 2015-VI, local catcher *leg*. [VNM]; 1♂, Sapa (1700 m), Lao Cai, 2017-V, local catcher *leg*. [SJH]. The holotype and three paratypes were deposited in Kunming Institute of Zoology (KIZ), Chinese Academy of Sciences, under the accession numbers KIZ0033834–KIZ0033836, KIZ0139644, and KIZ0139645. The remaining paratypes in Shao-Ji Hu’s private collection will be transferred to the main collection in Yunnan University in the future.

**Host plant:** *Mahonia duclouxiana* (Berberidaceae) (field observation by the first author in the type locality).

**Distribution:** Widely distributed from the central-eastern Yunnan plateau to Lao Cai and Ha Giang Province in N. Vietnam [5,46,50].

**Phenology:** Univoltine, the flight period of adults varies with latitude. In Ha Giang Province of Vietnam, it flies in May, while in N.E. Yunnan it occurs in June.

**Etymology:** The subspecific name of this new taxon was dedicated to Joseph F. Rock (1884–1962), a botanist, geographer, ethnologist, photographer, and writer who spent many years of his life researching, cataloguing, and reporting biodiversity and ethnic culture in Yunnan, Sichuan, Gansu, and Tibet of China.

##### *Aporia kuangtungensis kuangtungensis* Mell, 1935 **stat. nov.**

**Figure 21:**
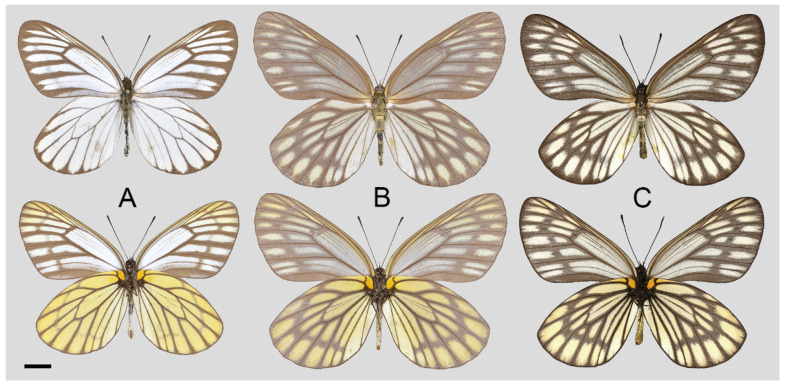
*Aporia kuangtungensis kuangtungensis* **stat. nov.** Mell, 1935; (**A**) male, (**B**,**C**) female; (**A**,**B**): Nanling, Guangdong, China (courtesy and © J. L. Chen); (**C**): Dawei Shan, Hunan, China (courtesy and © J. Q. Zhu); scale bar = 10 mm.

**Diagnostic characters (Figure 21):** Forewing length: male 42–48 mm (mean = 45.2 ± 1.6 mm, *n* = 15), female 44 mm (*n* = 2). Both wings broad and elongate, forewing apex more pointed. Upperside ivory white with thick black veins, postdiscal band, and termen, black veins near base thinner, forewing discocells with black fine lines and slightly peppered with dark scales, hindwing postdiscal band absent. Underside similar to upperside, forewing apical area and hindwing with strong dull-yellowish hue, hindwing postdiscal band present but discontinuous, black termen very narrow. Female ground colour greyish white with dust yellow hue, dark markings very thick (some individuals with extreme melanism could be much darker), both wings with a translucent texture, hindwing postdiscal band on both sides evident, continuous, and reaching vein 2A.

**Figure 22:**
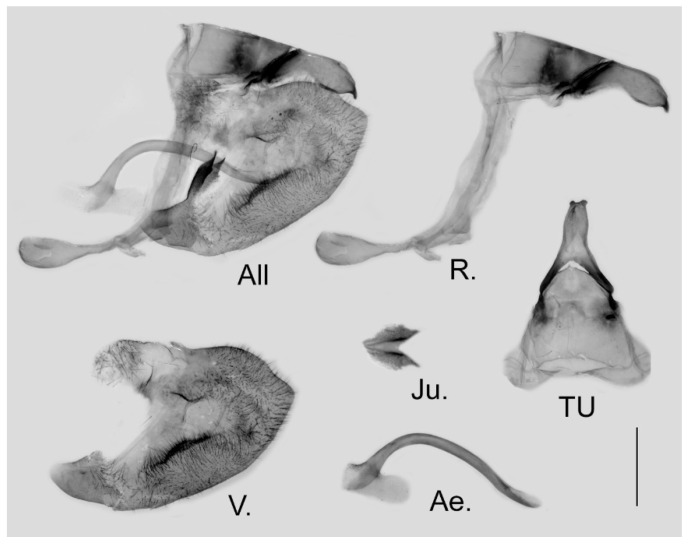
Male genitalia of *Aporia kuangtungensis kuangtungensis* **stat. nov.** Mell, 1935 from Nanling, Guangdong, China; All: entire male genitalia with left valve removed; R.: ring; TU: dorsal view of tegumen and uncus; V.: right valve; Ae.: aedeagus; Ju.: juxta; scale bar = 1 mm.

**Figure 23:**
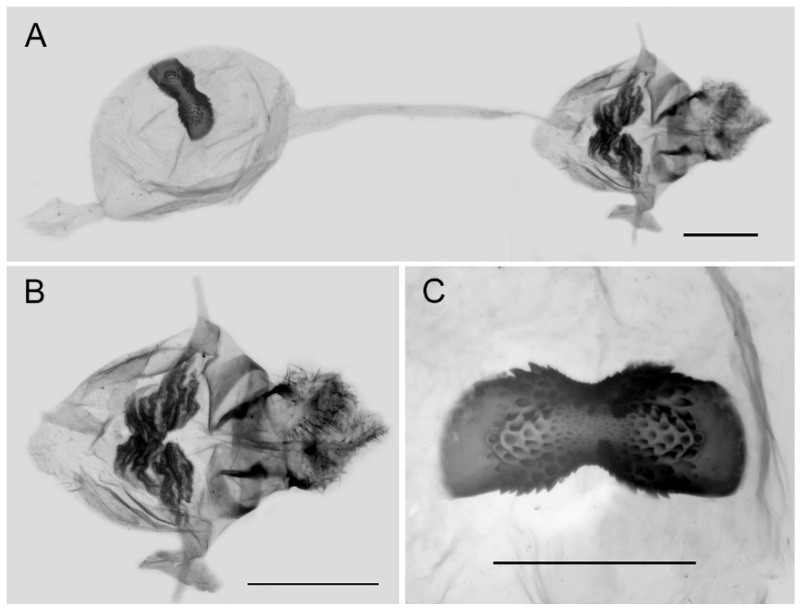
Female genitalia of *Aporia kuangtungensis kuangtungensis* **stat. nov.** Mell, 1935 from Dawei Shan, Hunan, China; (**A**): entire female genitalia; (**B**): enlarged ostium and sterigma; (**C**): enlarged signum; scale bars = 1 mm.

**Male and female genitalia: Similar to** those of the preceding subspecies (Figure 22 and Figure 23).

**Host plants:** *Mahonia bealei* (Berberidaceae) (J. L. Chen, pers. comm.).

**Distribution:** Found in Hunan, Guangdong and Guangxi of S. China.

##### *Aporia kuangtungensis pacifica* (Mell, 1943) **stat. rev.**

*M.*[*etaporia*] *largeteaui pacifica* Mell, 1943; Zool. Stuttgart, 16: 88; TL: “NW-Fukien (Kuatun…) und Chekiang (Tien mo shan, Wenchow)” [Guadun, Wuyi Shan, N.W. Fujian; Tianmu Shan and Wenzhou, Zhejiang, China] [14].

**Figure 24:**
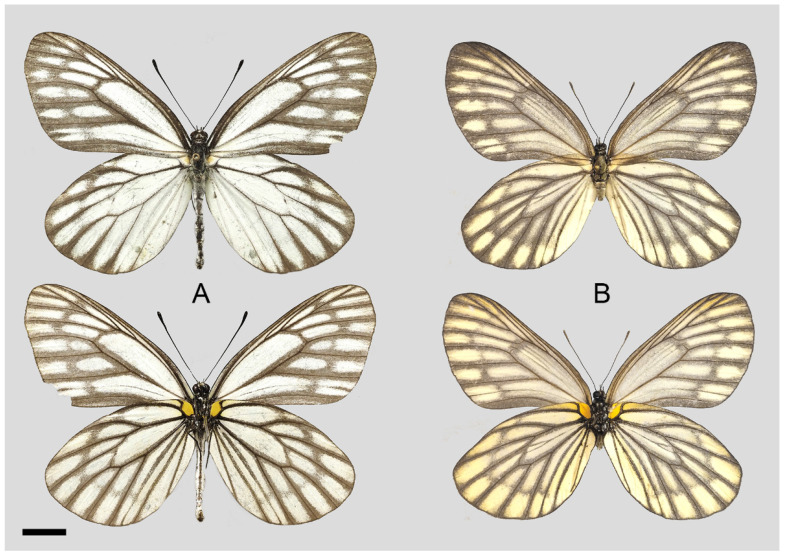
*Aporia kuangtungensis pacifica* (Mell, 1943) **stat. rev.**; A: male; B: female; (**A**): Wuyi Shan, Fujian, China (courtesy and © Z. J. Wu); (**B**): Baishanzu, Zhejiang, China; scale bar = 10 mm.

**Diagnostic characters (Figure 24):** Forewing length: male 44 mm (*n* = 1), female 40 mm (*n* = 1). Both wings broad and elongate, forewing apex more pointed. Upperside ivory white with thick black veins, postdiacal band, and termen, forewing discocell slightly peppered with dark scales, forewing postdiscal band present while hindwing postdiscal band often faint. Underside similar to upperside, hindwing postdiscal band evident, ends at vein 2A, lack of black termen. Female ground colour greyish white to pale dust yellow, dark markings similar to male but thicker, both wings with a translucent texture, hindwing postdiscal band on both sides more evident, usually continuous and reaching vein 2A.

**Figure 25:**
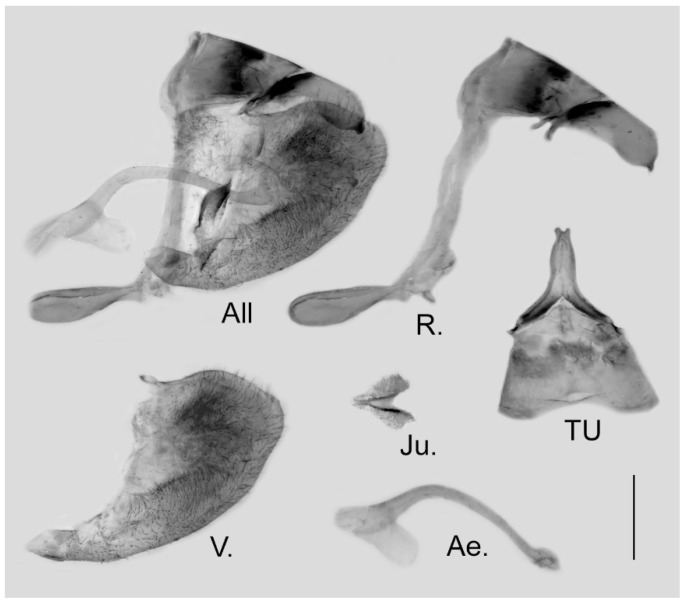
Male genitalia of *Aporia kuangtungensis pacifica* (Mell, 1943)**stat. rev.** from Wuyi Shan, Fujian, China; All: entire male genitalia with left valve removed; R.: ring; TU: dorsal view of tegumen and uncus; V.: right valve; Ae.: aedeagus; Ju.: juxta; scale bar = 1 mm.

**Figure 26:**
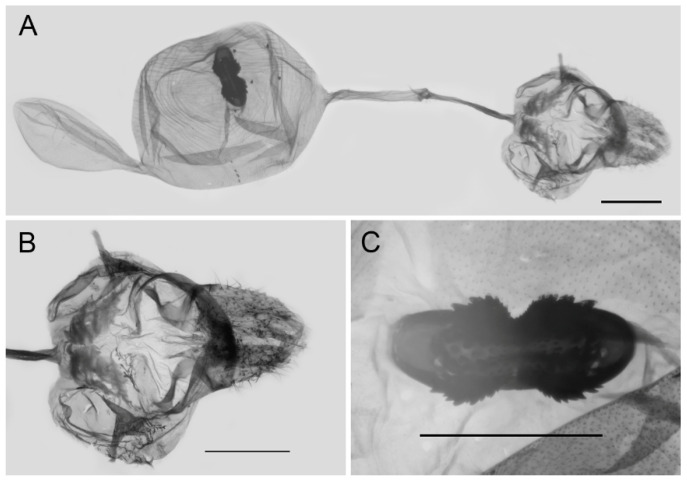
Female genitalia of *Aporia kuangtungensis pacifica* (Mell, 1943) **stat. rev.** from Baishanzu, Zhejiang, China; (**A**): entire female genitalia; (**B**): enlarged ostium and sterigma; (**C**): enlarged signum; scale bars = 1 mm.

**Male and female genitalia:** Similar to those of the preceding subspecies (Figure 25 and Figure 26).

**Host plants:** Uncertain, presumably *Mahonia* sp. (Berberidaceae).

**Distribution:** Found in Zhejiang of E. China.

##### *Aporia kuangtungensis cheni* Hsu & Chou, 1999 **stat. rev.**

*Aporia gigantea cheni* Hsu & Chou, 1999; Zool. Stud., 38: 223; TL: “TAIWAN: PINGTUNG Co., Wutai, Wutoushan” [Wutou Shan, Pingtung County, Taiwan Island, China] [16].

**Figure 27:**
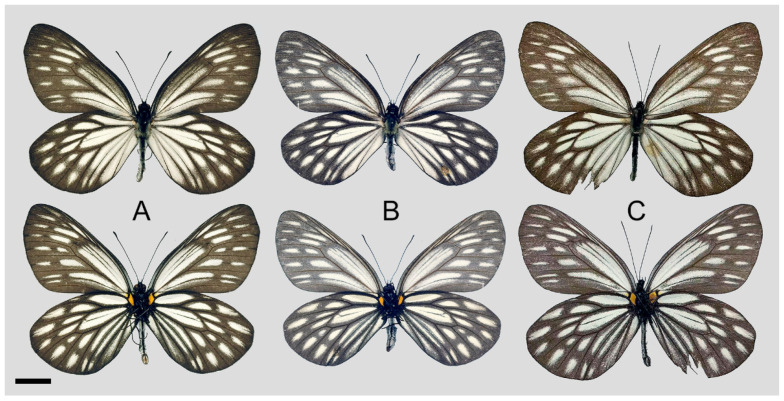
*Aporia kuangtungensis cheni* Hsu & Chou, 1999 **stat. rev.**, (**A**) male, (**B**) female, (**C**) male; (**A**,**B**): Taoyuan, Kaohsiung, Taiwan, China; (**C**): Wutai, Pingtung, Taiwan, China (courtesy and © Philip Y.-F. Lo); upperside above, underside below, scale bar = 10 mm.

**Diagnostic characters (Figure 27):** Forewing length: male 37–43 mm (mean = 41.5 ± 1.6 mm, *n* = 11), female 39–43 mm (mean = 40.5 ± 2.2 mm, *n* = 3). Both wings broad and round in male, while more elongate in female. Upperside greyish ivory-coloured with thick blackish veins and broad blackish margin and postdiscal band; discocells with bifid black lines. Underside similar, with a slight yellowish hue; hindwing veins in tornal area thicker than upperside. Female very similar to male, blackish markings paler.

**Figure 28:**
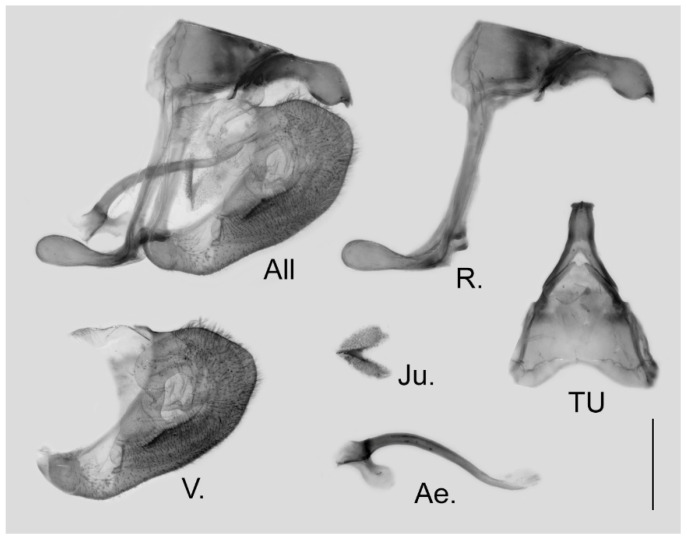
Male genitalia of *Aporia kuangtungensis cheni* Hsu & Chou, 1999 **stat. rev.** from Kao Hsiung, Taiwan Island, China; All: entire male genitalia with left valve removed; R.: ring; TU: dorsal view of tegumen and uncus; V.: right valve; Ae.: aedeagus; Ju.: juxta; scale bar = 1 mm.

**Figure 29:**
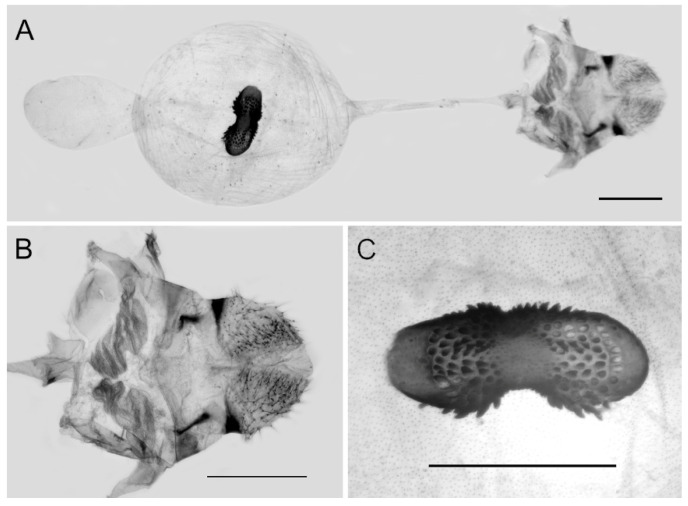
Female genitalia of *Aporia kuangtungensis cheni* Hsu & Chou, 1999 **stat. rev.** from Nanxi, Taitung, Taiwan Island, China; (**A**): entire female genitalia; (**B**): enlarged ostium and sterigma; (**C**): enlarged signum; scale bars = 1 mm.

**Male and female genitalia:** Similar to those of the preceding subspecies (Figure 28 and Figure 29).

**Host plants:** *Mahonia oiwakensis* (Berberidaceae) [12,16].

**Distribution:** China (Taiwan Island).

#### 3.2.5. *Aporia agathon* (Gray, 1831)

*Pieris Agathon* Gray, 1831; Zool. Miscell., 1: 33; TL: “Nepaul” [Nepal] [1].

**Figure 30:**
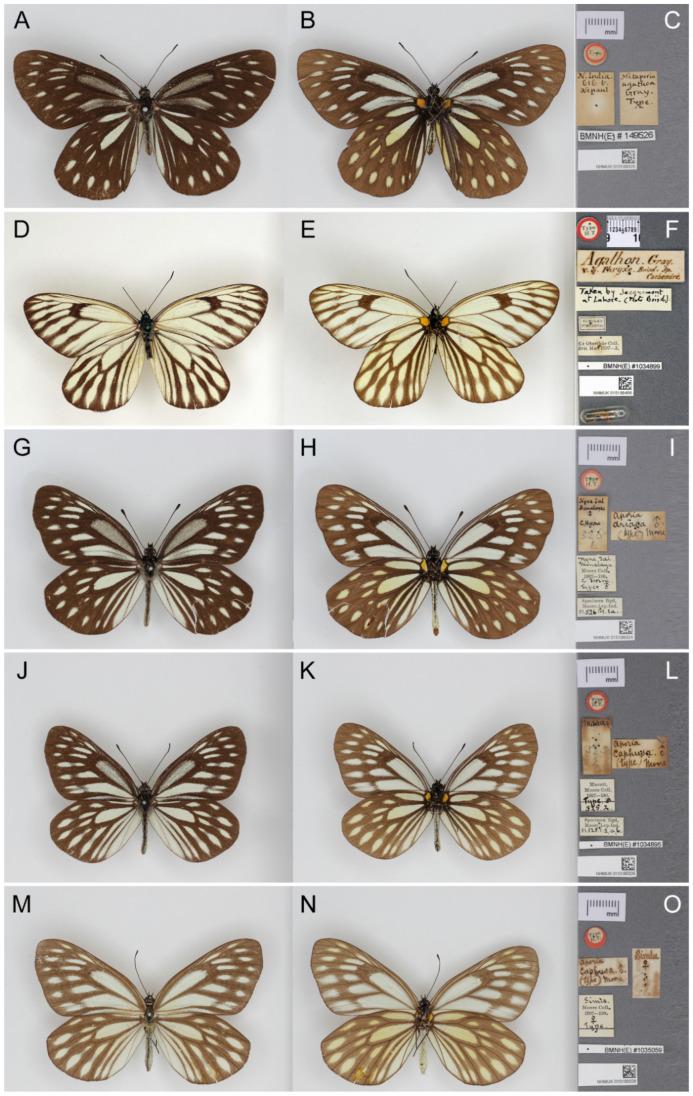
The Holotypes of various subspecies of *Aporia agathon* (Gray, 1831) deposited in the Natural History Museum, London, UK. (**A**–**C**): *Metaporia agathon* Gray, 1831, (**A**): upperside, (**B**): underside, (**C**): labels: round-type label with red circle and printed “Type”; rectangular label on the left with G. R. Gray’s handwritten “N. India./616 b./Nepaul”; rectangular label on the right (underside of the same previous label) with Gray’s handwritten “Metaporia agathon/Gray./Type”; the narrow rectangular label with printed “BMNH(E) # 149526”; the last (bottom) rectangular label bears an accession number and a QR code of the NHM; scale bar = 10 mm. (**D**–**F**): *Pieris Phryxe* Boisduval, 1836, (**D**): upperside, (**E**): underside, (**F**): labels: round-type label with red circle and printed “Type/HT”; the first rectangular label has, handwritten, “Agathon. Gray./v. ♀. Phryxe. Boisd. Sp./Cachemire”; the second rectangular label has, handwritten, “Taken by Jacquemont/at Lahore. (Type Boisd.)”; the third smallest rectangular label with black outline has, printed, “EX MUSÆO/D^ris.^ BOISDUVAL”; the fourth rectangular label has, printed, “Ex Oberthür Coll./Brit. Mus. 1927–3.”; the narrow rectangular label has, printed, “BMNH(E) #1034899”; the last rectangular label bears an accession number and a QR code of the NHM; the capsule at the bottom contains the detached abdomen; scale bar = 10 mm. (**G**–**I**): *Aporia Ariaca* Moore, 1872; (**G**): upperside; (**H**): underside; (**I**): labels—Round-type label with red circle and printed “Type/H.T.”; the rectangular label on the left with F. Moore’s handwritten “Nyne Tal/Himalayas/♂/C.Horne” and the other handwritten “562/1”; the underside on the right with F. Moore’s handwritten “Aporia Ariaca ♂/(Type) Moore”; the square label has “Moore Coll./1907–190.” printed, and handwritten “Nyne Tal./Himalaya.” above and “C. Horne./Type. ♂” below; the third rectangular label with printed “Specimen figd,/Moore, Lep.Ind./Pl.526(handwritten).f.1,1a.(handwritten)”; the last (bottom) rectangular label bears an accession number and a QR code of the NHM; scale bar = 10 mm. (**J**–**L**): *Aporia Caphusa* Moore, 1872 (male); (**J**): upperside; (**K**): underside; (**L**): labels: round-type label with red circle and printed “Type/H.T.”; the rectangular label on the left with F. Moore’s handwritten “Masuri” and the other handwritten “525/2”; the underside on the right with F. Moore’s handwritten “Aporia Caphusa, ♂/(Type) Moore”; the square label has, printed, “Masuri./Moore Coll./1907–190.” and handwritten “Type. ♂/525.2.”; the narrow rectangular label has, printed, “BMNH(E) #1034895”; the last (bottom) rectangular label bears an accession number and a QR code of the NHM; scale bar = 10 mm. (**M**–**O**): *Aporia Caphusa* Moore (female), 1872, (**M**): upperside, (**N**): underside, (**O**): labels: round-type label with red circle and printed “Type/H.T.”; the rectangular label on the left with F. Moore’s handwritten “Aporia Caphusa. ♂./(Type) Moore”; the underside on the right with F. Moore’s handwritten “Simla/♀”; the square label has, printed, “Moore Coll./1907–190.” and handwritten “Simla.” above it and “♀/Type.” below it; the narrow rectangular label with printed “BMNH(E) #1035059”; the last (bottom) rectangular label bears an accession number and a QR code of the NHM; scale bar = 10 mm. All type specimen photographs © Copyright Trustees of the Natural History Museum, used with permission under Creative Commons License 4.0 (https://creativecommons.org/licenses/by/4.0/). Any other use of this image, except for personal study, requires prior written consent of the housing institution.

**Distribution and subspecies:** Only found on the southern slope of the Himalayan Mountains. India: Uttarakhand, Himachal, Sikkim, Assam; Nepal: Kathmandu area; Bhutan; and China: S. Tibet. Three subspecies are recognised from the west to the east of their range (Figure 30).

##### *Aporia agathon phryxe* (Boisduval, 1836)

*Pieris Phryxe* Boisduval, 1836; Spec. gén. Lépid., 1: 446; TL: “Cachemire… Lahore, dans le Punchaûb” [Lahore, India] [6].
  *Metaporia Caphusa* Moore, 1872; Proc. zool. Soc. Lond., 1872: 564; TL: “N.W. Himalayas (Masuri, Simla, Kunawur)” [Mussoorie, Shimla and Kinnaur, Himachal Pradesh, N.W. India] [7].

**Diagnostic characters (Figure 30):** Forewing length: male 31–40 mm (mean = 36.9 ± 2.6 mm, *n* = 10), female 38–42 mm (*n* = 2). The most distinguishable character of ssp. *phryxe* is its paler wing colour with extreme to moderate reduced black markings on both sides, but the short blackish band at the end of forewing discocell is prominent.

**Figure 31:**
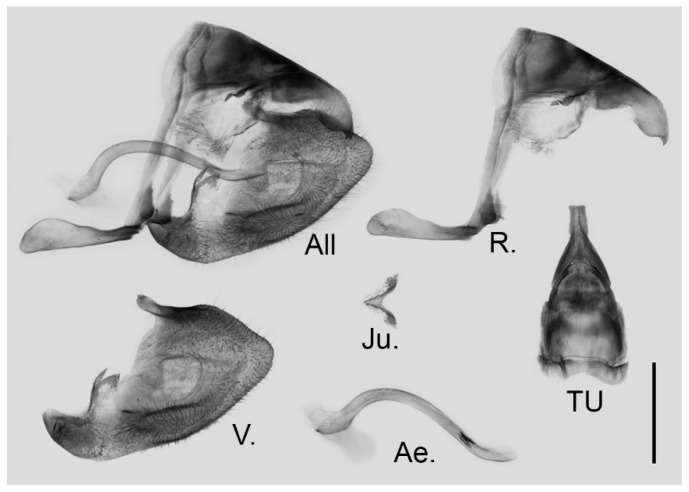
Male genitalia of *Aporia agathon phryxe* (Boisduval, 1836) from Mussoorie, India; All: entire male genitalia with left valve removed; R.: ring; TU: dorsal view of tegumen and uncus; V.: right valve; Ae.: aedeagus; Ju.: juxta; scale bar = 1 mm.

**Male genitalia (Figure 31):** Heavily sclerotized. Ring slender, anteriorly slightly curved near tegumen, angle with saccus perpendicular to slightly obtuse; uncus short and robust, dorsally broad at base, narrowed into a bifid tip; uncus laterally short and beak-like, dorsal margin smoothly curved ventrally, tip bifid and hooked; saccus of moderate length with expanded tip. Valve evidently elongate, heart-shaped, dorsal margin convex, ventral margin smooth, tip rounded, fovea large and mostly round, ventral margin of valve below fovea thickened. Aedeagus slender, curved with a trochanter at its ventral base. Juxta V-shaped with two arms separated from base.

**Figure 32:**
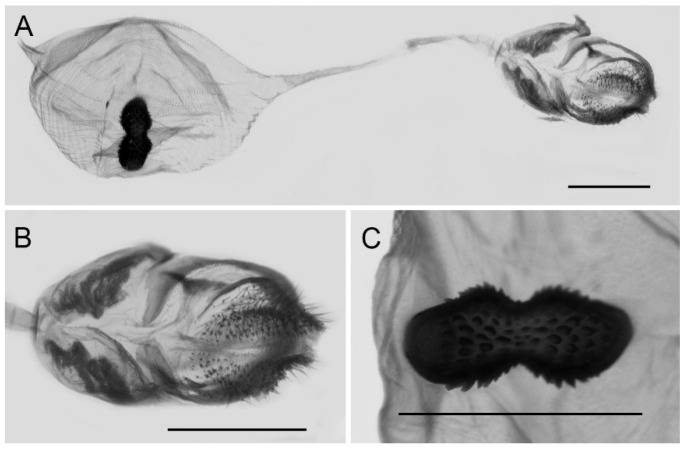
Female genitalia of *Aporia agathon phryxe* (Boisduval, 1836) from Mussoorie, India; (**A**): entire female genitalia; (**B**): enlarged ostium and sterigma; (**C**): enlarged signum; scale bars = 1 mm.

**Female genitalia (Figure 32):** See under ssp. *phryxe*.

**Distribution:** This subspecies has the widest distribution range in the Himalayas from N.W. India to S. Tibet of China.

##### *Aporia agathon agathon* (Gray, 1831)

**Diagnostic characters (Figure 30):** Forewing length: male 34–43 mm (mean = 37.7 ± 2.4 mm, *n* = 32), female 40–44 mm (mean = 42.3 ± 1.3 mm, *n* = 11). Darkest subspecies; ground colour blackish on both sides, markings ivory, forewing discocell heavily irrigated with dark scales on the upperside, hindwing discocell with black fine line.

**Figure 33:**
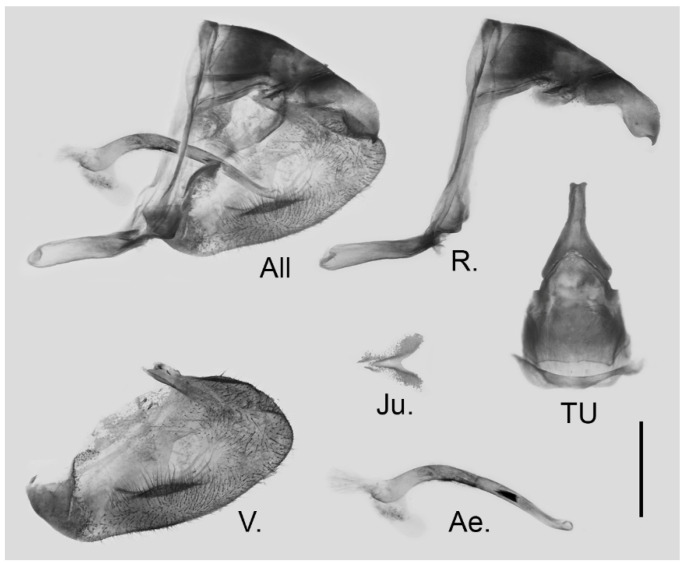
Male genitalia of *Aporia agathon agathon* (Gray, 1831) from Kathmandu valley, Nepal; All: entire male genitalia with left valve removed; R.: ring; TU: dorsal view of tegumen and uncus; V.: right valve; Ae.: aedeagus; Ju.: juxta; scale bar = 1 mm.

**Male genitalia and female genitalia: Similar to** those of ssp. *phryxe* (Figure 33).

**Distribution:** This subspecies is restricted to the mountains and valleys near Kathmandu of Nepal.

##### *Aporia agathon ariaca* (Moore, 1872) **reinst. stat. et stat. rev.**

*Metaporia Ariaca* Moore, 1872; Proc. zool. Soc. Lond., 1872: 564; TL: “Himalayas (Nynee Tal district) [Nainital, Uttarakhand, N. India]” [6].
  *Aporia agathon phryxe* f. *ariaca* (Moore, 1872) (Fruhstorfer [49]).
  *Aporia caphusa* Moore, 1872 (Evans [51]).
  *Aporia agathon ariaca* (Moore, 1872) (Talbot [52]).
  *Aporia agathon phryxe* (Boisduval, 1836) (Van Gasse [53]; see note for details).

**Diagnostic characters (Figure 30):** Forewing length: male 37–38 mm (mean = 37.4 ± 0.5 mm, *n* = 5), female 39–42 mm (mean = 40.8 ± 1.3 mm, *n* = 4). Ivory markings on both wings larger than ssp. *agathon*, less dark scales in the forewing discocell on the upperside, forewing apical area and hindwing with more evident yellow hue on the underside.

**Figure 34:**
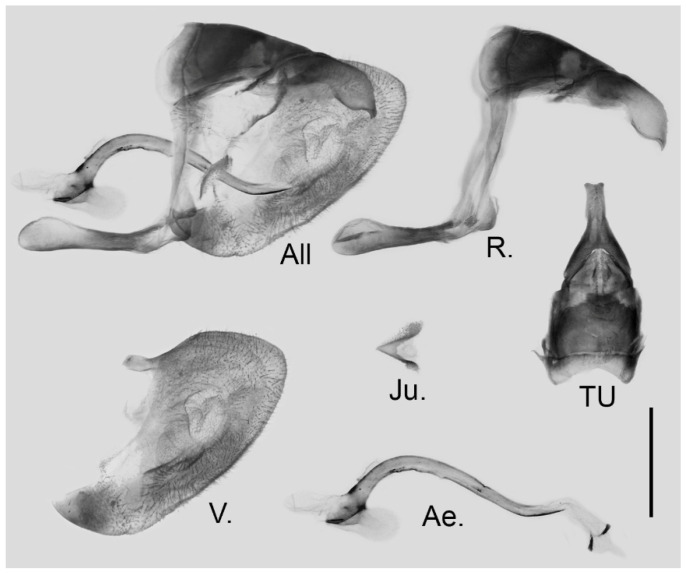
Male genitalia of *Aporia agathon ariaca* (Moore, 1872) **reinst. stat. et stat. rev.** from Tashiyangse, Bhutan; All: entire male genitalia with left valve removed; R.: ring; TU: dorsal view of tegumen and uncus; V.: right valve; Ae.: aedeagus; Ju.: juxta; scale bar = 1 mm.

**Figure 35:**
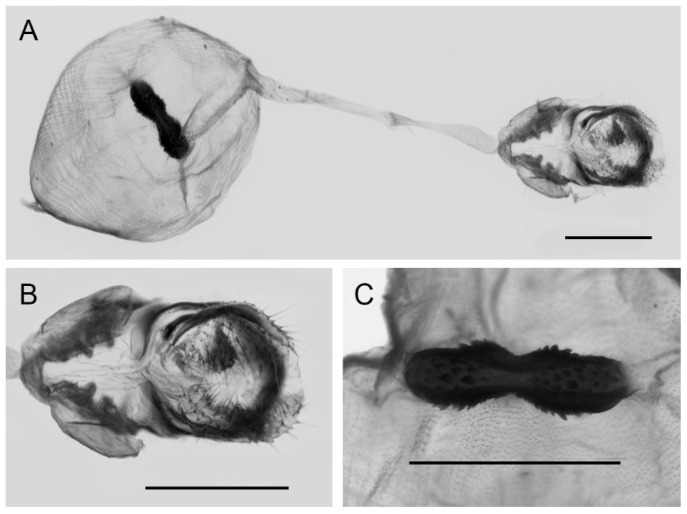
Female genitalia of *Aporia agathon ariaca* (Moore, 1872) **reinst. stat. et stat. rev.** from Tashiyangse, Bhutan; (**A**): entire female genitalia; (**B**): enlarged ostium and sterigma; (**C**): enlarged signum; scale bars = 1 mm.

**Note:** Subspecies *caphusa* (Moore, 1872) is sympatric with ssp. *phryxe* but with a darker appearance. Van Gasse [53] listed the latter under ssp. *phryxe* without further explanation. Based on the large volume of photographs listed by Kunte et al. [54] and specimens examined with help of Kotaro Saito (Japan), there are intermediate forms between typical *phryxe* and *caphusa* phenotypes, and the recognised populations shared identical DNA barcodes in our molecular analysis. Hence, the present study agrees with Van Gasse [53] and treats ssp. *caphusa* as a synonym of ssp. *phryxe*. The situation of ssp. *ariaca* is more complicated. It was described as a species, and Fruhstorfer [49] downgraded it as a form of *phryxe*. Evans [51] treated it as a synonym of *caphusa*, while Talbot [52] treated as a valid subspecies, which was followed by subsequent literature like those of Della Bruna et al. [55] and Della Bruna, et al. [5]. Van Gasse [53] treated *ariaca* as a synonym of *phryxe* based on Fruhstofer’s opinion. However, our molecular analysis confirmed the distinct subspecies identity of the Bhutanese population designated under ssp. *ariaca* in the BOLD database (Table 1; Figure 2 and Figure 3). The type locality of *ariaca* is Nainital, Uttarakhand, N. India, which falls into the range of ssp. *phryxe*. One possibility is that the opinion of Evans is correct and the Bhutanese population does not have a name yet. Due to a lack of sufficient material from across the Himalayas, the authors chose to keep the Bhutanese population as ssp. *ariaca* until future evidence could elucidate it.

**Male genitalia and female genitalia:** Similar to those of ssp. *phryxe* and ssp. *agathon* (Figure 34 and Figure 35).

**Distribution:** N.W. India, Nepal and Bhutan.

#### 3.2.6. *Aporia omotoi* Yoshino, 2003 **stat. nov.**

**Figure 36:**
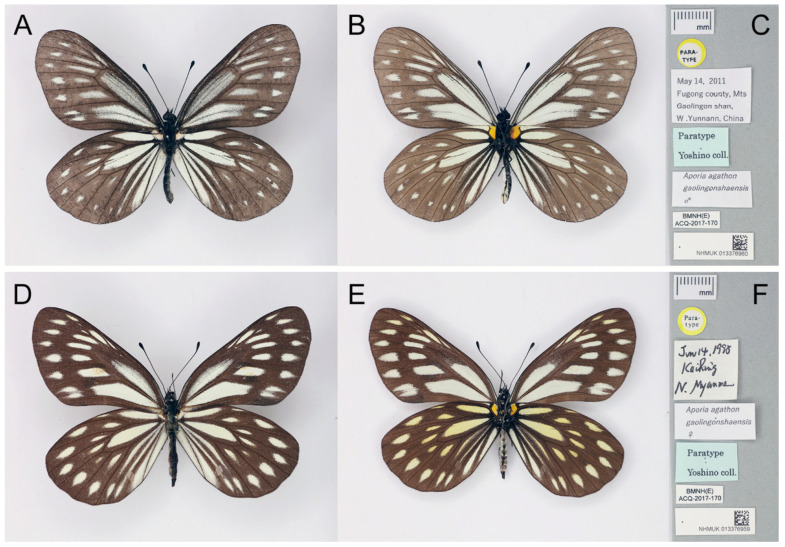
The paratypes of *Aporia agathon gaolingongshanensis* Yoshino, 2015, deposited in the Natural History Museum, London, UK; (**A**–**C**): male; (**A**): upperside, (**B**): underside, (**C**): labels: round-type label with yellow circle and printed “Para-type”, the first rectangular label has, printed, “May 14 2011/Fugong county Mts/Gaolingon shan [Gaoligong Shan]/W. Yunnann [Yunnan], China”, the second (light blue) rectangular label with printed “Paratype/Yoshino coll.”; the third rectangular label has, printed, “*Aporia agathon/gaolingongshaensis* ♂.”; the fourth rectangular label has, printed, “BMNH(E) ACQ-2017-170”; the last (bottom) rectangular label bears an accession number and a QR the code of the NHM; scale bar = 10 mm. (**D**–**F**): female; (**D**): upperside; (**E**): underside, (**F**): labels: round-type label with yellow circle and printed “Para-type”, the first rectangular label with K. Yoshino’s handwritten “Jun 14 1998/KaiRing [Kachin]/N. Myanmar”, the second rectangular label has, printed, “*Aporia agathon/gaolingongshaensis* ♀.”; the third (light blue) rectangular label has, printed, “Paratype/Yoshino coll.”; the fourth rectangular label has, printed, “BMNH(E) ACQ-2017-170”; the last (bottom) rectangular label bears an accession number and a QR code of the NHM; scale bar in mm. Type specimen photographs © Copyright Trustees of the Natural History Museum, used with permission under Creative Commons License 4.0 (https://creativecommons.org/licenses/by/4.0/). Any other use of this image, except for personal study, requires prior written consent of the housing institution.

*Aporia agathon omotoi* Yoshino, 2003; Futao, 43: 6, f. 1–4; TL: “Zhondian county… North Yunnan, China” [Zhongdian, N.W. Yunnan, China] [9].
  *Aporia agathon gaolingonshanensis* Yoshino, 2015; Butterfly Science, 2: 25, f. 5–8; TL: “Mts. Gaolingonshan, W. Yunnan” [Gaoligong Shan, W. Yunnan, China] [8]. **syn. nov.** (Figure 36).
  *Aporia agathon gaolingonshaensis* Yoshino, 2015; Butterfly Science, 2: 26, Table 1, f. 5–8 (caption). [IOS].

**Figure 37:**
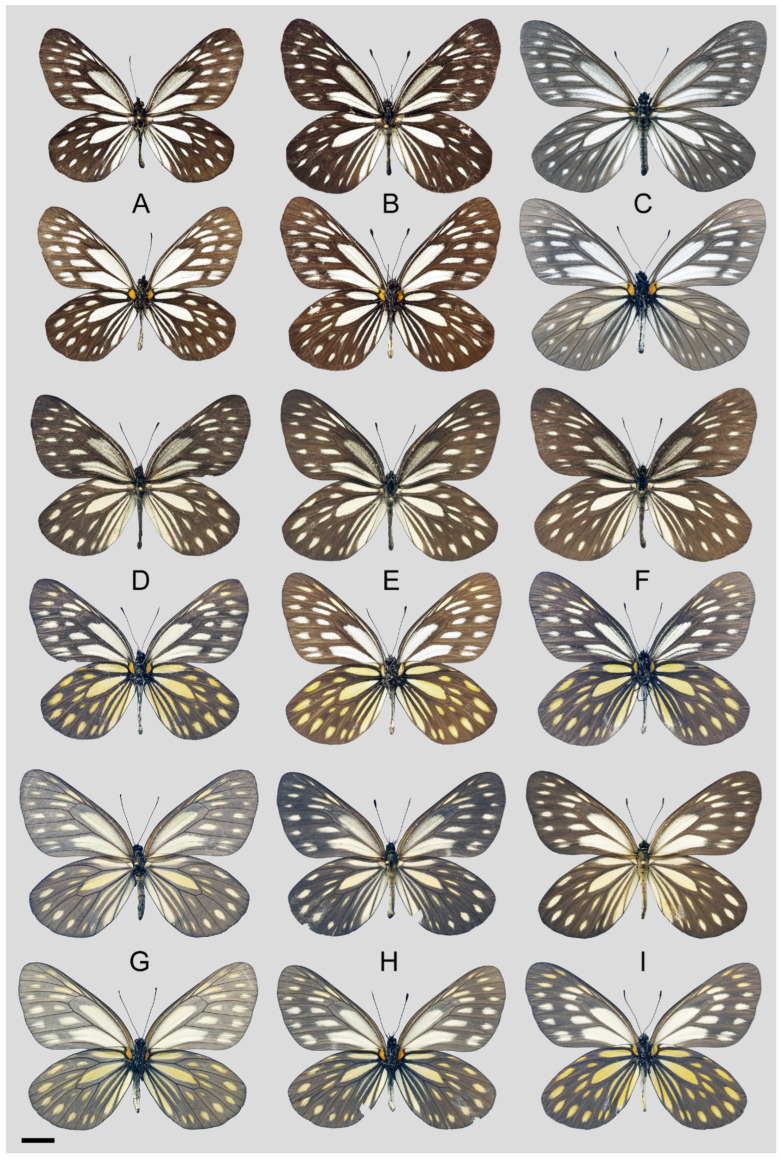
*Aporia omotoi* Yoshino, 2003 **stat. nov.**, (**A**,**B**,**D**–**F**) male, (**C**,**G**–**I**) female; (**A**,**B**): Gongshan, Yunnan, China; (**C**): Longling, Yunnan, China; (**D**): Zhongdian, Yunnan, China; (**E**,**F**): Yulong, Yunnan, China; (**G**): Weixi, Yunnan, China; (**H**): Zhongdian, Yunnan, China; (**I**): Yulong, Yunnan, China; upperside above, underside below, scale bar = 10 mm.

**Diagnostic characters (Figure 37):** Forewing length: male 37–42 mm (mean = 39.2 ± 1.5 mm, *n* = 16), female 39–43 mm (mean = 41.0 ± 1.4 mm, *n* = 11). Both wings broad and rounded, seldom elongate. Upperside blackish with white to ivory-coloured markings, discocells without black lines, discocell on forewing upperside irrigated with dark scales. Underside brownish, markings on hindwing and forewing apical area ivory-coloured or slightly tinged with yellow, while other markings on forewing whitish. Female similar to male but paler with a translucent texture.

**Figure 38:**
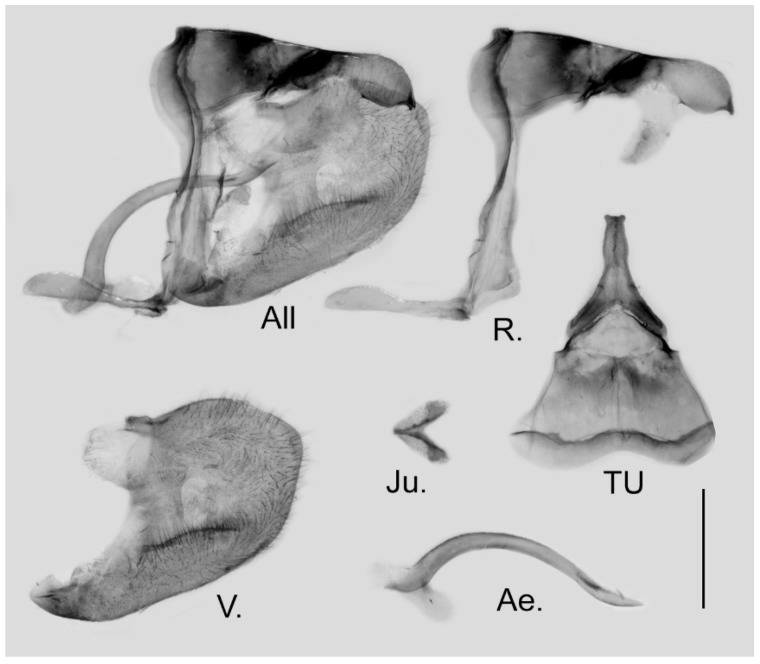
Male genitalia of *Aporia omotoi* Yoshino, 2003 **stat. nov.** from Yulong Xueshan, Yulong, Yunnan, China; All: entire male genitalia with left valve removed; R.: ring; TU: dorsal view of tegumen and uncus; V.: right valve; Ae.: aedeagus; Ju.: juxta; scale bar = 1 mm.

**Male genitalia (Figure 38):** Heavily sclerotized. Ring slender, anteriorly curved near tegumen; angle to saccus perpendicular or obtuse; uncus dorsally broad at base, gradually narrowed into a shaft with swollen median section and outwardly bifid tip; uncus laterally beak-like, dorsal margin straight at basal third and smoothly curved ventrally in the remaining two-thirds, tip bifid and hooked; saccus long and narrow. Valve elongate, approximately rectangular, dorsal margin slightly convex, ventral margin smooth, tip rounded, fovea large and oval, ventral margin of valve below fovea thickened. Aedeagus slender, curved with a trochanter at its ventral base. Juxta narrow V-shaped with two arms gradually separating, tip broader.

**Figure 39:**
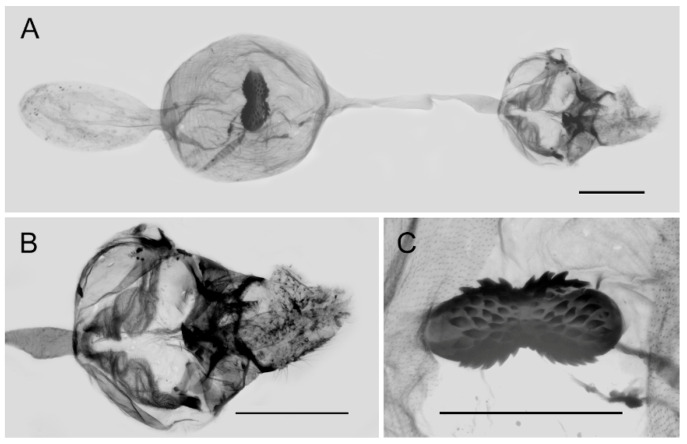
Female genitalia of *Aporia omotoi* Yoshino, 2003 **stat. nov.** from Yulong Xueshan, Yulong, Yunnan, China; (**A**): entire female genitalia; (**B**): enlarged ostium and sterigma; (**C**): enlarged signum; scale bars = 1 mm.

**Female genitalia (Figure 39):** Papillae anales elongate, tip rounded, covered with dense setae. Apophyses posteriors in long rod shape, apophyses anteriores in short conical shape. Lamella antevaginalis connects a pair of hairy, double-folded sterigma, with the basal part smaller triangular and the distal part larger blunt-tipped, lamella postvaginalis slightly sclerotized. Ductus bursae tubular and membranous, rather slender, but broader near base. Corpus bursae oval with appendix bursae attached anteriorly, signum broad and bowknot-shaped, mostly covered with coarse spines except for two tips.

**Host plants:** Uncertain, presumably *Berberis* spp. (Berberidaceae).

**Distribution and subspecies:** China: W. to N.W. Yunnan, S. Tibet; Myanmar: Kachin State. Two subspecies are recognised.

**Note:** In the original description of *Aporia agathon omotoi*, Yoshino [9] compared *omotoi* with ‘*Aporia agathon bifurcata*’ from Tengchong, W. Yunnan, which evidently was *Aporia omotoi gaolingonshanensis*. The DNA barcode data from BOLD System used in this study contained two specimens collected from Tongme (Tibet, China) (Table 1), one being *A. agathon phryxe* and the other being *A. omotoi* (Figure 2 and Figure 3). The sympatry of the two taxa further supported *A. omotoi* being a distinct species.

#### 3.2.7. *Aporia largeteaui* (Oberthür, 1881)

**Figure 40:**
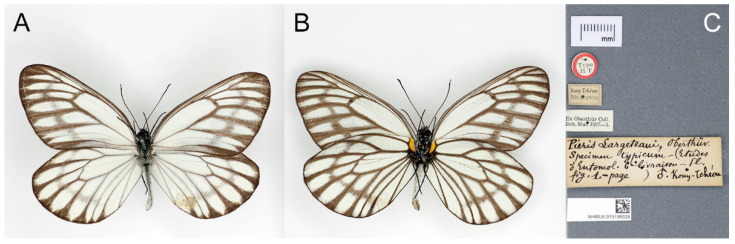
The Holotype of *Pieris Largeteaui* Oberthür, 1881 (male), deposited in the Natural History Museum, London, UK; (**A**): upperside, (**B**): underside, (**C**): labels: round-type label with red circle and printed “Type/HT”; the first (smallest) rectangular label with black outline and printed “Kouy-Tchéou/Abbé Largeteau“, the second rectangular label has, printed, “Ex Oberthür Coll./Brit. Mus. 1927–3”, the third (biggest) rectangular label with C. Oberthür’s handwritten “Pieris Largeteaui, Oberthür./Specimen Typicum—(Etudes d’Entomol. 6—livraison—rl, fig. 1.—page) ♂. Kouy-Tchéou”, and the last (bottom) rectangular label bears an accession number and a QR code of the NHM; scale bar = 10 mm. Type specimen photographs © Copyright Trustees of the Natural History Museum, used with permission under Creative Commons License 4.0 (https://creativecommons.org/licenses/by/4.0/). Any other use of this image, except for personal study, requires prior written consent of the housing institution.

*Pieris Largeteaui* Oberthür, 1881; Étud. d’Ent., 6: 12, pl. 7, f. 1; TL: “Kouy-Tchéou” [Guizhou, China] (Figure 40) [2].

**Distribution and subspecies:** Widely distributed in Central, Southern, and Eastern China, as well as N. Vietnam, with five recognised subspecies.

##### *Aporia largeteaui lemoulti* Bernardi, 1944 **stat. rev.**

*Aporia* (*Metaporia*) *Agathon Le Moulti* Bernardi, 1944; Misc. Ent., 41: 75; TL: “Ning-Yuen-Fou (Szechwan)” [Xichang, W. Sichuan. China] [4].
  *Aporia lemoulti* Bernardi, 1944 (Della Bruna et al. [55]).
  *Aporia morishitai* Chou, 1994; Monographia Rhop. Sinensium: 754; TL: Miyi, Sichuan, China [56].

**Figure 41:**
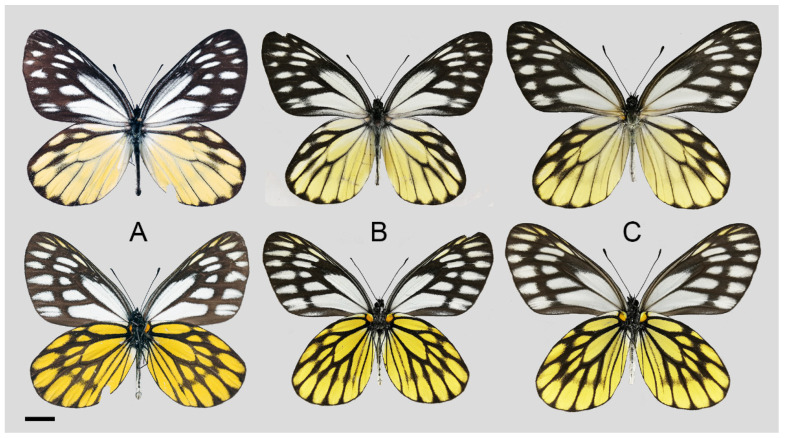
*Aporia largeteaui lemoulti* Bernardi, 1944 **stat. rev.**; A–B male (courtesy and © (**A**): Y.-F. Li; (**B**): H.-H. Zhang); (**C**) female (courtesy and © H.-H. Zhang); Puwei, Miyi, Sichuan, China; upperside above, underside below, scale bar = 10 mm.

**Diagnostic characters (Figure 41):** Forewing length: male 41–42 mm (*n* = 2), female 43–44 mm (*n* = 2). Both wings broad and slightly elongate. Upperside forewing white, with thick black veins, postdiscal band, and termen; veins near base less darkened, discocell slightly peppered with dark scales but without black lines; hindwing lemon yellow with black veins and termen, postdiscal band only poorly indicated, veins in and near tornal area less darkened. Underside similar to upperside; hindwing postdiscal band more evident but still discontinuous, veins in tornal area blackish. Female similar to male but paler with a translucent texture, hindwing postdiscal band on the underside continuous.

**Figure 42:**
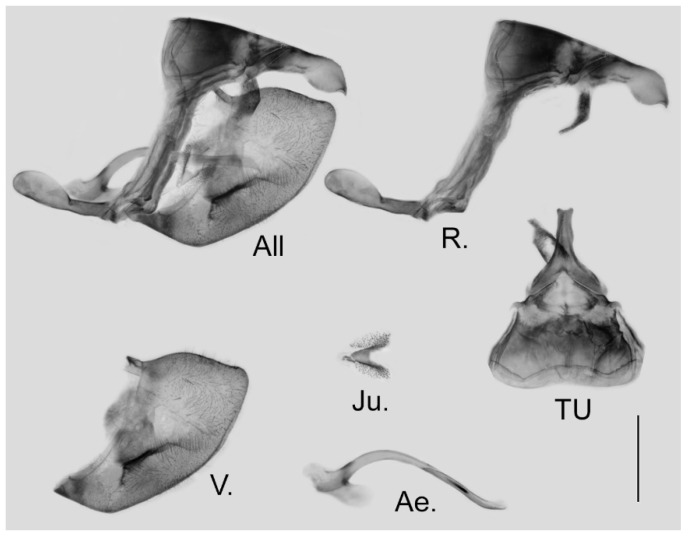
Male genitalia of *Aporia largeteaui lemoulti* Bernardi, 1944 **stat. rev.** from Puwei, Miyi, Sichuan, China; All: entire male genitalia with left valve removed; R.: ring; TU: dorsal view of tegumen and uncus; V.: right valve; Ae.: aedeagus; Ju.: juxta; scale bar = 1 mm.

**Male genitalia (Figure 42):** Heavily sclerotized. Ring slender, anteriorly slightly curved near tegumen, angle to saccus obtuse; uncus dorsally broad at base, evidently narrowed into shaft with slightly swollen median section and outwardly bifid tip; uncus laterally beak-like, dorsal margin straight at basal half and smoothly curved ventrally in the remaining half, tip bifid and hooked; saccus medium sized. Valve elongate, approximately heart-shaped, dorsal margin slightly convex, ventral margin smooth, tip rounded but evident, fovea large and oval, ventral margin of valve below fovea thickened. Aedeagus slender, curved with a trochanter at its ventral base. Juxta narrow, V-shaped, with two arms separate near base; tip broader.

**Figure 43:**
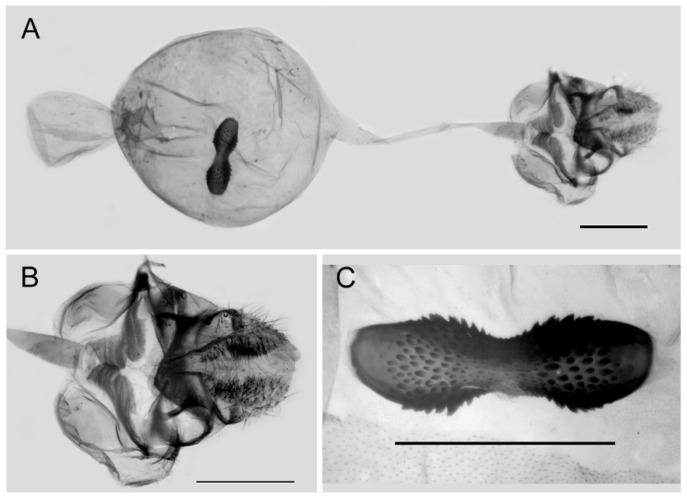
Female genitalia of *Aporia largeteaui lemoulti* Bernardi, 1944 **stat. rev.** from Puwei, Miyi, Sichuan, China; (**A**): entire female genitalia; (**B**): enlarged ostium and sterigma; (**C**): enlarged signum; scale bars = 1 mm.

**Female genitalia (Figure 43):** Papillae anales short, tip rounded, covered with dense setae. Apophyses posteriors in long rod shape, apophyses anteriores in short conical shape. Lamella antevaginalis connects a pair of hairy double-folded sterigma, with the basal part shorter broader and the distal part longer finger-like, lamella postvaginalis slightly sclerotized. Ductus bursae tubular and membranous, rather slender, but broader near base. Corpus bursae oval with appendix bursae attached anteriorly, signum bowknot-shaped, nearly entirely covered with spines (spines in the central part much finer) except for two tips.

**Host plants:** *Mahonia fortunei* (Berberidaceae).

**Distribution:** Confined to the eastern margin of the Hengduan Mountains in southern Sichuan Province.

##### *Aporia largeteaui largeteaui* (Oberthür, 1881)

**Figure 44:**
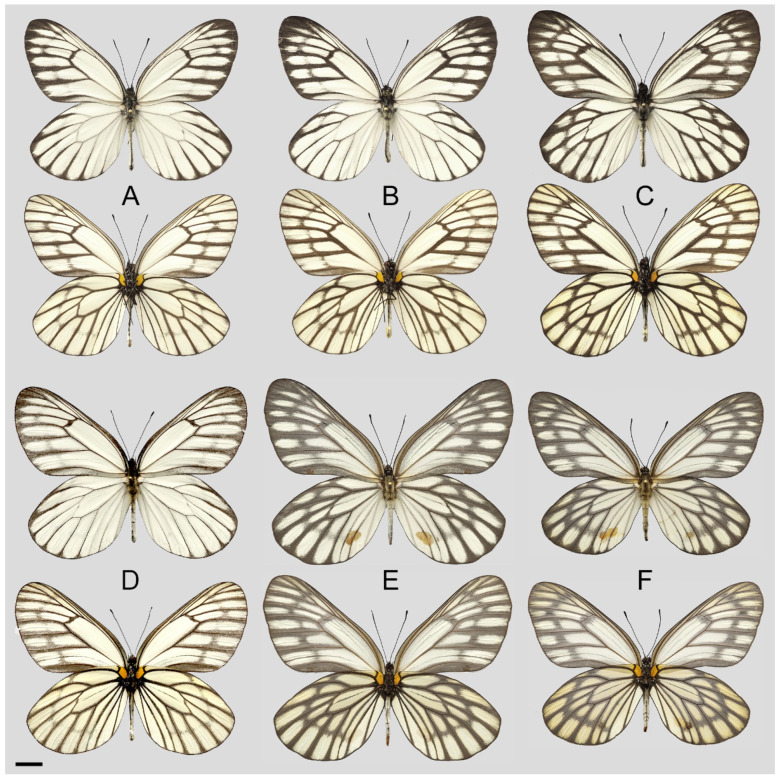
*Aporia largeteaui largeteaui* (Oberthür, 1881), (**A**–**D**) male, (**E**,**F**) female; (**A**–**C**): Baokang, Hubei, China; (**D**–**F**): Weixin, Yunnan, China; upperside above, underside below, scale bar = 10 mm.

**Diagnostic characters (Figure 44):** Forewing length: male 42–48 mm (mean = 45.2 ± 1.6 mm, *n* = 15), female 50–57 mm (mean = 53.8 ± 2.0 mm, *n* = 15). Both wings broad and rounded, hindwing apex angulate. Upperside pale white, veins in basal half not darkened, while in distal half gradually darkened and thickened towards black termen; postdiscal band only indicated, forewing discocell clean or with only fine black lines, hindwing veins not darkened, but tinged with blackish near termen, postdiscal band absent or poorly indicated. Underside similar to upperside, forewing apical area and hindwing with pale yellowish hue, postdiscal band more evident. Female ground colour greyish white, dark markings similar to male but much thicker, both wings with a translucent texture, discocells with blackish lines, postdiscal band on both sides more evident and continuous.

**Figure 45:**
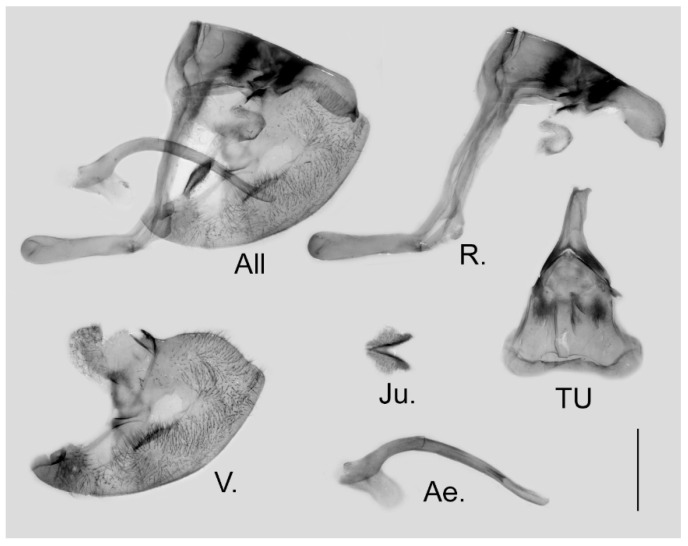
Male genitalia of *Aporia largeteaui largeteaui* (Oberthür, 1881) from Zunyi, Guizhou, China; All: entire male genitalia with left valve removed, R.: ring, TU: dorsal view of tegumen and uncus, V.: right valve, Ae.: aedeagus, Ju.: juxta; scale bar = 1 mm.

**Male genitalia (Figure 45):** Heavily sclerotized. Ring slender, anteriorly convex near tegumen, angle with saccus perpendicular to obtuse; uncus dorsally broad at base, gradually narrowed into a shaft with bifid tip; uncus laterally beak-like, dorsal margin gradually and smoothly curved ventrally, tip bifid and hooked; saccus moderate sized with broad tip. Valve slightly elongate, heart shaped, both dorsal and ventral margins smooth, tip rounded, fovea large and oval, ventral margin of valve below fovea thickened. Aedeagus slender, curved with a trochanter at its ventral base. Juxta V-shaped with two arms separated immediately at base, tip broader.

**Figure 46:**
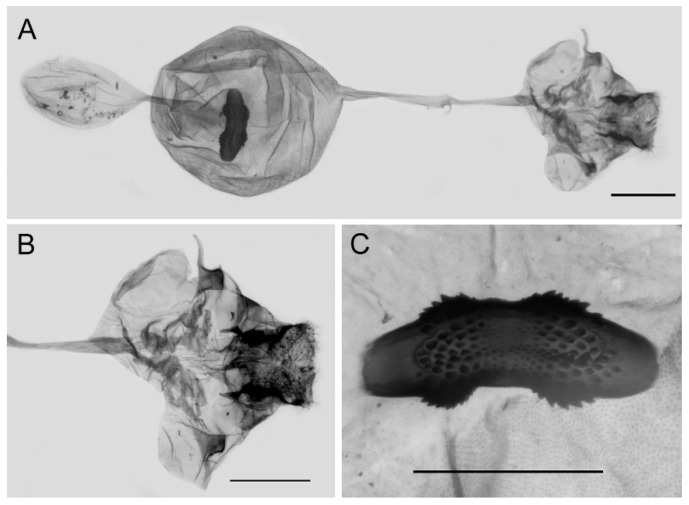
Female genitalia of *Aporia largeteaui largeteaui* (Oberthür, 1881) from Weixin, Yunnan, China; (**A**): entire female genitalia; (**B**): enlarged ostium and sterigma; (**C**): enlarged signum; scale bars = 1 mm.

**Female genitalia (Figure 46):** Papillae anales slightly elongate, tip rounded, covered with dense setae. Apophyses posteriors in long rod shape, apophyses anteriores in short conical shape. Lamella antevaginalis connects a pair of hairy double-folded sterigma, with the basal part shorter broader and the distal part longer triangulate, lamella postvaginalis slightly sclerotized. Ductus bursae tubular and membranous, rather slender, but broader near base. Corpus bursae oval with appendix bursae attached anteriorly, signum bowknot-shaped, nearly entirely covered with coarse spines except for central part.

**Host plants:** *Mahonia bealei* [57] (Berberidaceae).

**Distribution:** Widely distributed in N.E. Yunnan, Guizhou, Chongqing and Hubei of W. China.

##### *Aporia largeteaui fanjinensis* Yoshino, 1997 **stat. rev.**

*Aporia largeteaui fanjinensis* Yoshino, 1997; Neo Lepid., 2 (2): 2, f. 13–14; TL: “Mt. Fanjinshan, Tongren county, Guizhou prov., China” [Fanjing Shan, Tongren, Guizhou, China] [15].

**Figure 47:**
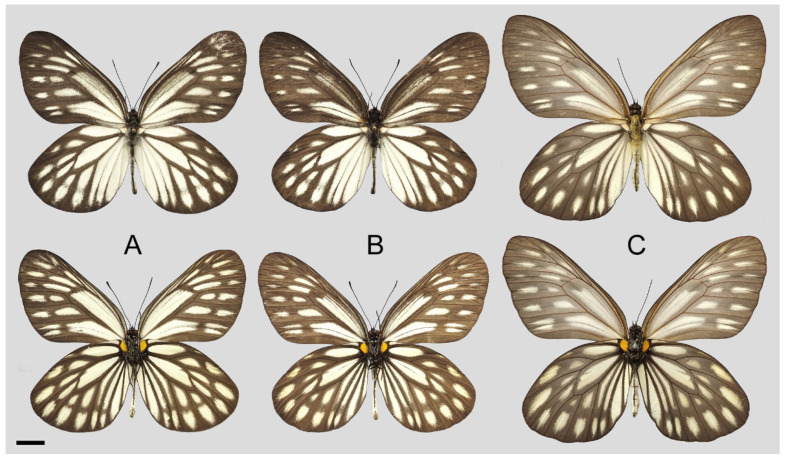
*Aporia largeteaui fanjinensis* Yoshino, 1997 **stat. rev.**; (**A**,**B**) male; (**C**) female; (**A**–**C**): Jinfo Shan, Chongqing, China; upperside above, underside below, scale bar = 10 mm.

**Diagnostic characters (Figure 47):** Forewing length: male 43–48 mm (mean = 46.1 ± 1.7 mm, *n* = 14), female 49–55 mm (*n* = 2). Both wings broad and less elongate, forewing apex round. Upperside ivory white with very thick black veins, postdiscal band, and termen, black veins, discocells with black lines, forewing discocell peppered with dark scales, hindwing postdiscal band often discontinuous and ending at vein M_3_ or CuA_1_. Underside similar to upperside, postdiscal bands more evident and discontinuous, ending at vein 2A, black termen broad. Female ground colour greyish white, dark markings similar to male but thicker and strongly suffused with dark scales, both wings with a translucent texture, postdiscal band of hindwing on both sides more evident, usually continuous and reaching vein 2A.

**Figure 48:**
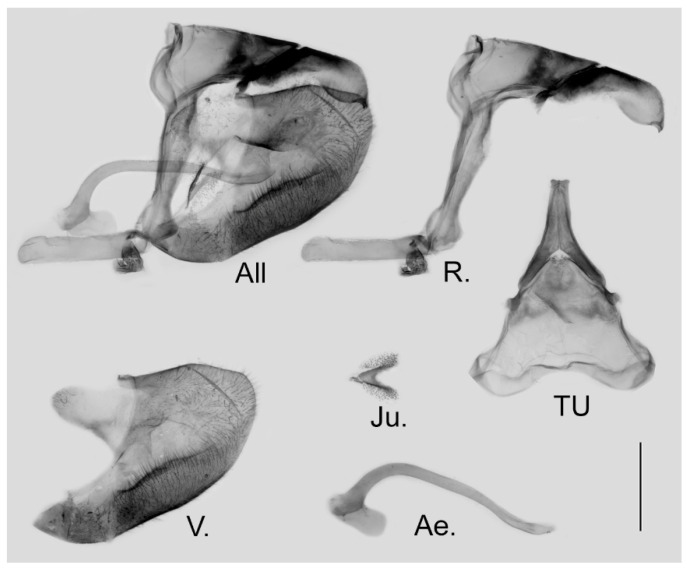
Male genitalia of *Aporia largeteaui fanjinensis* Yoshino, 1997 **stat. rev.** from Jinfo Shan, Chongqing, China; All: entire male genitalia with left valve removed; R.: ring; TU: dorsal view of tegumen and uncus; V.: right valve; Ae.: aedeagus; Ju.: juxta; scale bar = 1 mm.

**Figure 49:**
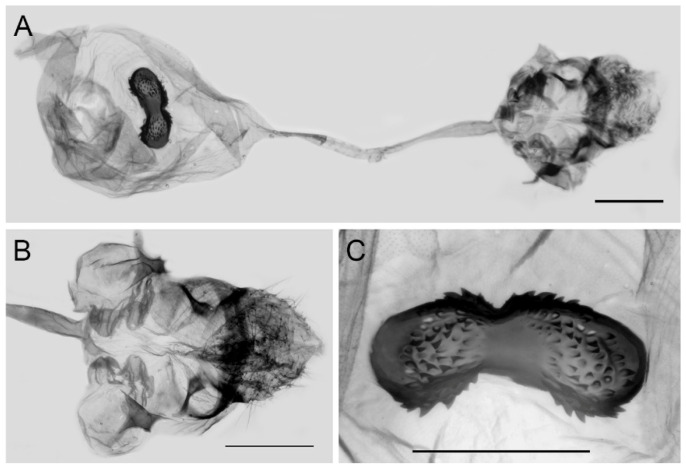
Female genitalia of *Aporia largeteaui fanjinensis* Yoshino, 1997 **stat. rev.** from Jinfo Shan, Chongqing, China; (**A**): entire female genitalia, (**B**): enlarged ostium and sterigma, (**C**): enlarged signum; scale bars = 1 mm.

**Male and female genitalia:** similar to those of the nominate subspecies (Figure 48 and Figure 49).

**Host plants:** Unknown, presumably species of *Mahonia* sp. (Berberidaceae).

**Distribution:** This subspecies is restricted to the Dalou Mountains in Guizhou and Chongqing, W. China.

##### *Aporia largeteaui schmackeri* (Mell, 1943)

*M.*[*etaporia*] *largeteaui schmackeri* Mell, 1943; Zool. Stuttgart, 36: 88; TL: “Auf Etikett nur “China” angegeben… mit ziemlicher Sicherheit Kuling…” [presumably Guling, Lu Shan, N. Jiangxi, China] [14].

**Figure 50:**
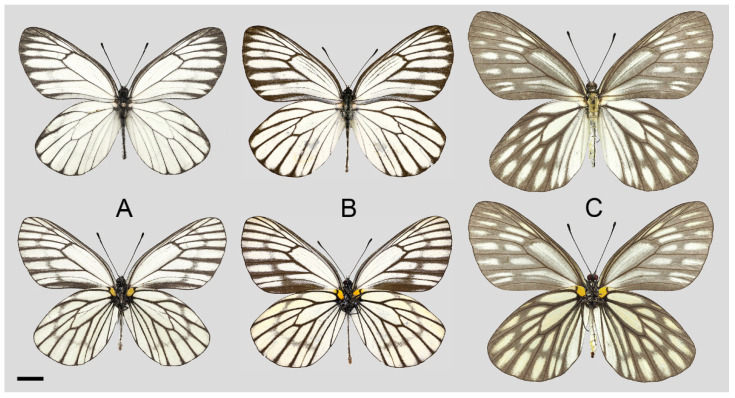
*Aporia largeteaui schmackeri* (Mell, 1943); (**A**,**B**) male, (**C**) female; (**A**,**C**): Wuyi Shan, Fujian, China (courtesy and © Z.-J. Wu), (**B**): Qingliang Feng, Zhejiang, China (courtesy and © W.-W. Mao); upperside above, underside below, scale bar = 10 mm.

**Diagnostic characters (Figure 50):** Forewing length: male 45–47 mm (mean = 46.3 ± 1.2 mm, *n* = 3), female 52 mm (*n* = 1). Both wings broad and moderately elongate. Upperside ivory white with black veins (in some individuals thicker), postdiscal band, and termen, black veins near base thinner, discocells with or without black fine lines, hindwing postdiscal band often invisible. Underside similar to upperside, forewing apical area and hindwing usually without yellowish hue, forewing and hindwing postdiscal band rather faint and discontinuous, black termen narrower. Female ground colour greyish white, dark markings much thicker, both wings with a translucent texture, postdiscal band on both sides evident and continuous and reaching vein 2A.

**Figure 51:**
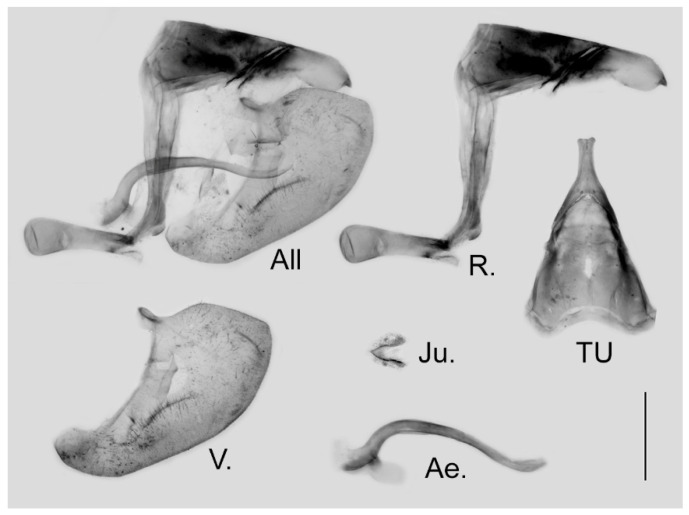
Male genitalia of *Aporia largeteaui schmackeri* (Mell, 1943) from Wuyi Shan, Fujian, China; All: entire male genitalia with left valve removed; R.: ring; TU: dorsal view of tegumen and uncus; V.: right valve; Ae.: aedeagus; Ju.: juxta; scale bar = 1 mm.

**Figure 52:**
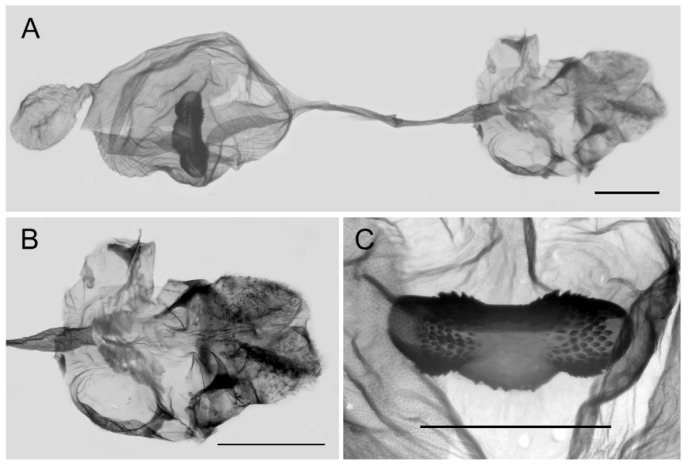
Female genitalia of *Aporia largeteaui schmackeri* (Mell, 1943) from Wuyi Shan, Fujian, China; (**A**): entire female genitalia; (**B**): enlarged ostium and sterigma; (**C**): enlarged signum; scale bars = 1 mm.

**Male and female genitalia:** similar to those of the nominate subspecies (Figure 51 and Figure 52).

**Host plants:** Unknown, presumably species of *Mahonia* sp. (Berberidaceae).

**Distribution:** Found in Jiangxi, Fujian and Zhejiang of S.E. to E. China.

##### *Aporia largeteaui gigantea* Koiwaya, 1993 **stat. nov.**

*Aporia gigantea* Koiwaya, 1993; Stud. Chinese Butt., 2: 91; TL: “Emei Shan, Sichuan” [Emei Shan, W. Sichuan. China] [3].

**Figure 53:**
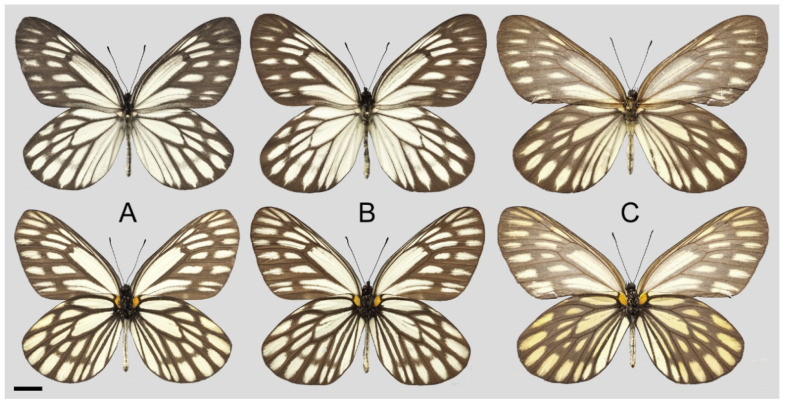
*Aporia largeteaui gigantea* Koiwaya, 1993 **stat. nov.**; (**A**,**B**) male, (**C**) female; (**A**–**C**): Ya’an, Sichuan, China; upperside above, underside below, scale bar = 10 mm.

**Diagnostic characters (Figure 53):** Forewing length: male 39–49 mm (mean = 45.6 ± 3.1 mm, *n* = 12), female 45–53 mm (mean = 50.3 ± 2.9 mm, *n* = 8). Both wings broad and moderately elongate, forewing apex more pointed. Upperside ivory white with thick black veins, postdiscal band, and termen; black veins near base thinner, discocells with black fine lines, forewing discocell slightly peppered with dark scales, hindwing postdiscal band often discontinuous and ending at vein M_3_ or CuA_1_. Underside similar to upperside, forewing apical area and hindwing with yellowish hue, hindwing postdiscal band more evident and discontinuous, ends at vein 2A, black termen narrower. Female ground colour greyish white to pale-dust yellow, dark markings similar to male but thicker, both wings with a translucent texture, hindwing postdiscal band on both sides more evident, usually continuous and reaching vein 2A.

**Figure 54:**
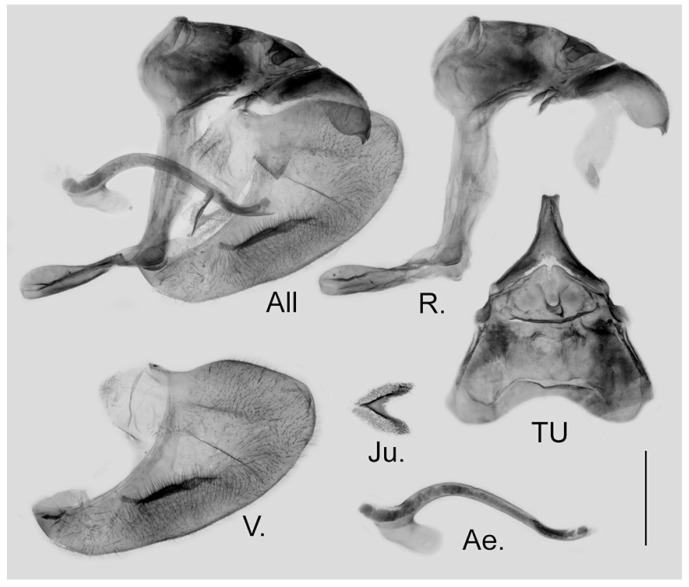
Male genitalia of *Aporia largeteaui gigantea* Koiwaya, 1993 **stat. nov.** from Shangli, Ya’an, Sichuan, China; All: entire male genitalia with left valve removed; R.: ring; TU: dorsal view of tegumen and uncus; V.: right valve; Ae.: aedeagus; Ju.: juxta; scale bar = 1 mm.

**Figure 55:**
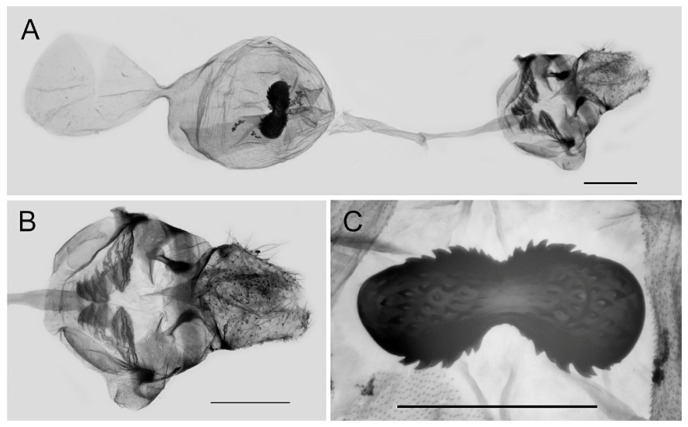
Female genitalia of *Aporia largeteaui gigantea* Koiwaya, 1993 **stat. nov.** from Shangli, Ya’an, Sichuan, China; (**A**): entire female genitalia; (**B**): enlarged ostium and sterigma; (**C**): enlarged signum; scale bars = 1 mm.

**Male and female genitalia:** similar to those of the nominate subspecies (Figure 54 and Figure 55).

**Host plants:** *Mahonia bealei* [16] (Berberidaceae).

**Distribution:** Found from the eastern margin of the Hengduan Mountains in W. Sichuan, W. China.

## 4. Discussion

The taxonomic confusion underpinning the *Aporia agathon* group had been detected by some previous research involving other Pieridae or *Aporia* species [18,58], but is now systematically explained for the first time by this research. Judging from other research regarding the cryptic species in *Aporia*, as well as other butterfly groups with the Hengduan Mountains as their diversity centre [19,59,60], it is highly likely that other species groups in the genus *Aporia* might also contain cryptic species that require future investigation to clarify.

Our analysis showed that one major factor causing taxonomic confusion in the *A. agathon* group is the variation in melanism among taxa and populations. The most typical case is that of *A. gigantea*, described by Koiwaya [3], which adopted the heavy black marking as one of its key diagnostics. Although the original description claimed differences in male genitalia, it is the problematic ‘*A. largeteaui*’ used for comparison which caused this difference. During literature review, the first author discovered that the ‘*A. largeteaui*’ from N. Sichuan used by Koiwaya was actually an *A. kuangtungensis yufeii* described in this research [3]. The latter taxon is smaller than *A. largeteaui*, while its colour and wing pattern are very similar to the type specimen of the nominate *A. largeteaui* in Oberthür’s original description (and its figure) [2]. This confusion also extended to Mell’s descriptions of several subsequent taxa under the name *A. largeteaui*, which actually consists of two species, with his ssp. *kuangtungensis* being a distinct species (*A. kuangtungensis*), ssp. *pacifica* being a subspecies of *A. kuangtungensis*, and ssp. *schmackeri* being a subspecies of *A. largeteaui*. In more recent literature, this confusion also affected Della Bruna et al. [5], in which the nominate subspecies of *A. largeteaui* was also *A. kuangtungensis yufeii.* The most reliable morphological characteristics separating *A. largeteaui* and *A. kuangtungensis* stat. nov. are male and female genitalia; the shape of uncus in lateral view and the structure of sterigma in ventral view are good diagnostic characters. In general, the lateral view of uncus of *A. kuangtungensis* is more angulate and the ventral view of its sterigma contains a pair of almost-parallel structures, while in *A. largeteaui*, the lateral view of uncus is much smoother and the ventral view of sterigma contains two separate parts (Figure 16, Figure 17, Figure 19, Figure 20, Figure 22, Figure 23, Figure 25, Figure 26, Figure 28, Figure 29, Figure 42, Figure 43, Figure 45, Figure 46, Figure 48, Figure 49, Figure 51, Figure 52, Figure 54 and Figure 55). The signum of female genitalia can also provide some clues, such as the shape and teeth arrangement. Other genital parts such as the valve, juxta, and aedeagus are less useful due to variation in shapes. The other confusion, caused by melanism, occurs between ssp. *phryxe* and ssp. *caphusa* of *A. agathon*, as the former taxon represents an extremely pale phenotype and the latter one has more black markings, but transitional individuals can also be found [54]. Their conspecific identity, revealed by our analysis, properly explains their sympatric distribution mapped in Della Bruna, et al. [5].

The yellow tinge on the hindwing underside is the other variable character in this species group. The extreme cases are *moltrechti*, which has been misidentified as *Delias taiwana*, and *lemoulti*, as a separate species [4,48], due to their bright yellow on the hindwing underside compared to other species in the same group. However, other species, such as *A. bifurcata*, *A. kuangtungensis*, *A. agathon*, and *A. omotoi* also exhibit variation in the yellow tinge on the hindwing underside when a long series of specimens and live photographs [54] were examined. For these species, the yellow tinge is less related to their subspecies or populations, but taxonomic confusions have also been caused by it—for instance, in the case of ssp. *gaolingonshanensis* and ssp. *sapaensis* of the previous *A. agathon* [8,11]. Our analysis showed very limited genetic divergence between *lemoulti* and other subspecies of *A. largeteaui* (Figure 4; Table S1), and their genitalic structures are also considerably similar. Given such evidence, the present research treated *lemoulti* as a subspecies of *A. largeteaui* rather than a distinct species, regardless of its very unique wing pattern and colour configuration. In contrast, the genetic divergence between *moltrechti* and its morphological ally, *A. agathon*, is larger than 5% (Figure 4; Table S1), and it was positioned in a different clade separated by other taxa (Figure 2 and Figure 3). Based on this, it deserves distinct species status rather than that of a subspecies of *A. agathon*. Similar situations were also found for *japfuensis*, *bifurcata*, and *omotoi*.

In this research, we also confirmed that *A. largeteaui* and *A. kuangtungensis* occupy different elevational ranges in the same mountain where their distribution seems sympatric. The direct evidence can be seen in Wuyi Shan (Fujian, China), in our sampling sites, where *A. largeteaui schmackeri* was collected from lower elevation (1000 m), while *A. kuangtungensis pacifica* was collected from a higher elevation near the mountain top (2100 m) (Table 1). Other indirect evidence is in N.E. Yunnan, where *A. kuangtungensis josephi* was collected from ~2000 m elevation range, while *A. largeteaui largeteaui* was collected from a 980 m elevation range during our expeditions to that area. This finding implies that *Aporia* has likely undergone historical elevational divergence. Similar phenomena were also reported in butterflies like *Papilio maackii* vs. *P. syfanius* [61] and *Papilio verityi* vs. *P. everesti* [62,63] in recent years. In N. Vietnam, specimens with *largeteaui*-like appearance were also reported as sympatric from Ha Giang by Monastyrskii [17], though future research must identify those populations carefully to determine their exact taxonomic identity, or detect whether a potential hybrid zone exists between the two species.

*Aporia* is a group of pierid butterflies with interesting phylogenetic and phylogeographic properties. To date, their evolutionary process is still poorly understood, although Kanoh et al. [64] developed a theory using wing markings (melanism, Y-streaks, etc.). However, based on the findings of our research and other available publications tackling this topic [18,65], wing markings are of limited usage in deciphering the evolutionary history of sibling *Aporia* species. Future research is very necessary to finally answer this question with a broader and deeper sampling effort, comprehensive morphological data, and genomic evidence. For the variation in melanism and yellow coloration, transcriptome technology with captive rearing of representative species could be a future direction to elucidate their mechanisms.

## 5. Conclusions

Pierid species of the *Aporia agathon* group were analysed using mitogenomic data; the results show *A. japfuensis* stat. nov., *A. bifurcata* stat. nov., *A. moltrechti* stat. rev., *A. kuangtungensis* stat. nov., and *A. omotoi* stat. nov. should be elevated to full species, while *lemoulti*, *gigantea*, and *fanjinensis* should be treated as subspecies of *A. largeteaui* syn. nov., stat. nov. Two subspecies of *A. kuangtungensis* stat. nov., namely *yufeii* **ssp. nov.** and *josephi* **ssp. nov.** are described for the first time. *A. kuangtungensis* stat. nov. and *A. largeteaui* are two complex species with multiple subspecies across their distribution range, and the wing patterns exhibit obvious variation. The authors believe such variation is the main cause of taxonomic confusions in this group, and future research using more samples and molecular evidence are necessary to answer this question.

## Data Availability

The gene sequence data supporting the findings of this study are openly available in the GenBank of NCBI at https://www.ncbi.nlm.nih.gov/genbank/. The DNA barcode (*cox1*) sequences are under the following accession numbers: PQ450495–PQ450507; the associated Bio-Project and Bio-Sample numbers are PRJNA967499 and SAMN44189788–SAMN44189800. The mitogenomes are under the following accession numbers: PQ481730–PQ481757; the associated Bio-Project number is PRJNA901130. The Zoobank registration of this article is: lsid:zoobank.org:pub:0318B776-ECD7-49B6-8F30-BC0458B99651.

## Supplementary Materials

The following supporting information can be downloaded at https://www.mdpi.com/article/10.3390/insects15120988/s1: Figure S1: The Bayesian phylogenetic tree of all recognised taxa, with outgroups included. Values at nodes indicate the posterior probability values; Figure S2: The IQ-tree of all recognised taxa, with outgroups included. Values at nodes indicate the boot-strap values with 10,000 iterations; Table S1: The Kimura two-parameter (K2P) genetic distances between all taxa.
