# Supplementary figures and images for: Disentangling Taxonomic Confusions in the Aporia agathon Group Using Mitochondrial Genomic Data (Lepidoptera: Pieridae)
Source: Insects. 2024 Dec 12;15(12):988. doi: 10.3390/insects15120988 (PMC11678737; doi:10.3390/insects15120988)

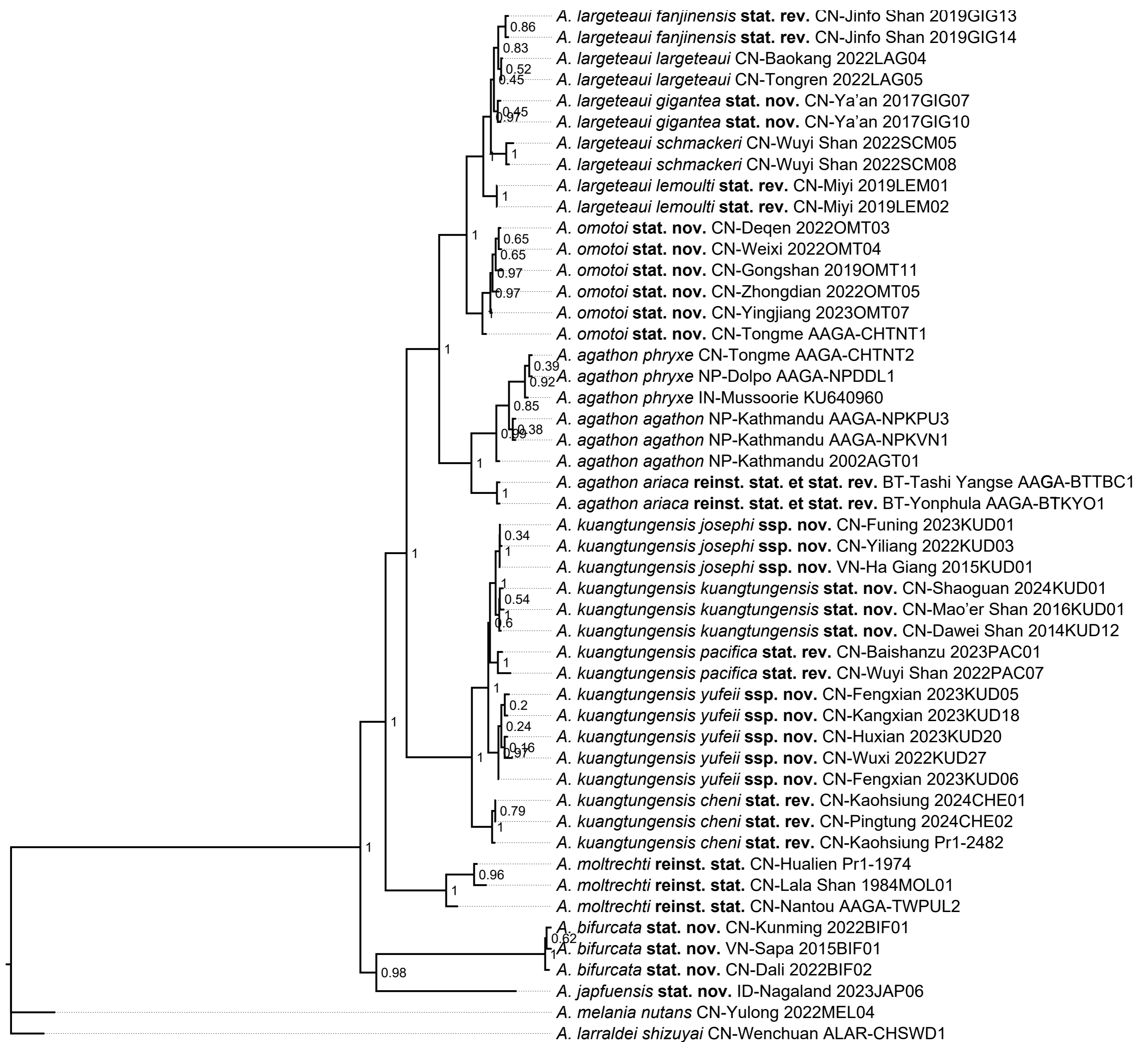

0.02

Supplement: Supplementary file 1 [file insects-15-00988-s001.zip › Suppl, Mat/Figure S1. The Bayesian phylogenetic tree (nodevalue).pdf]

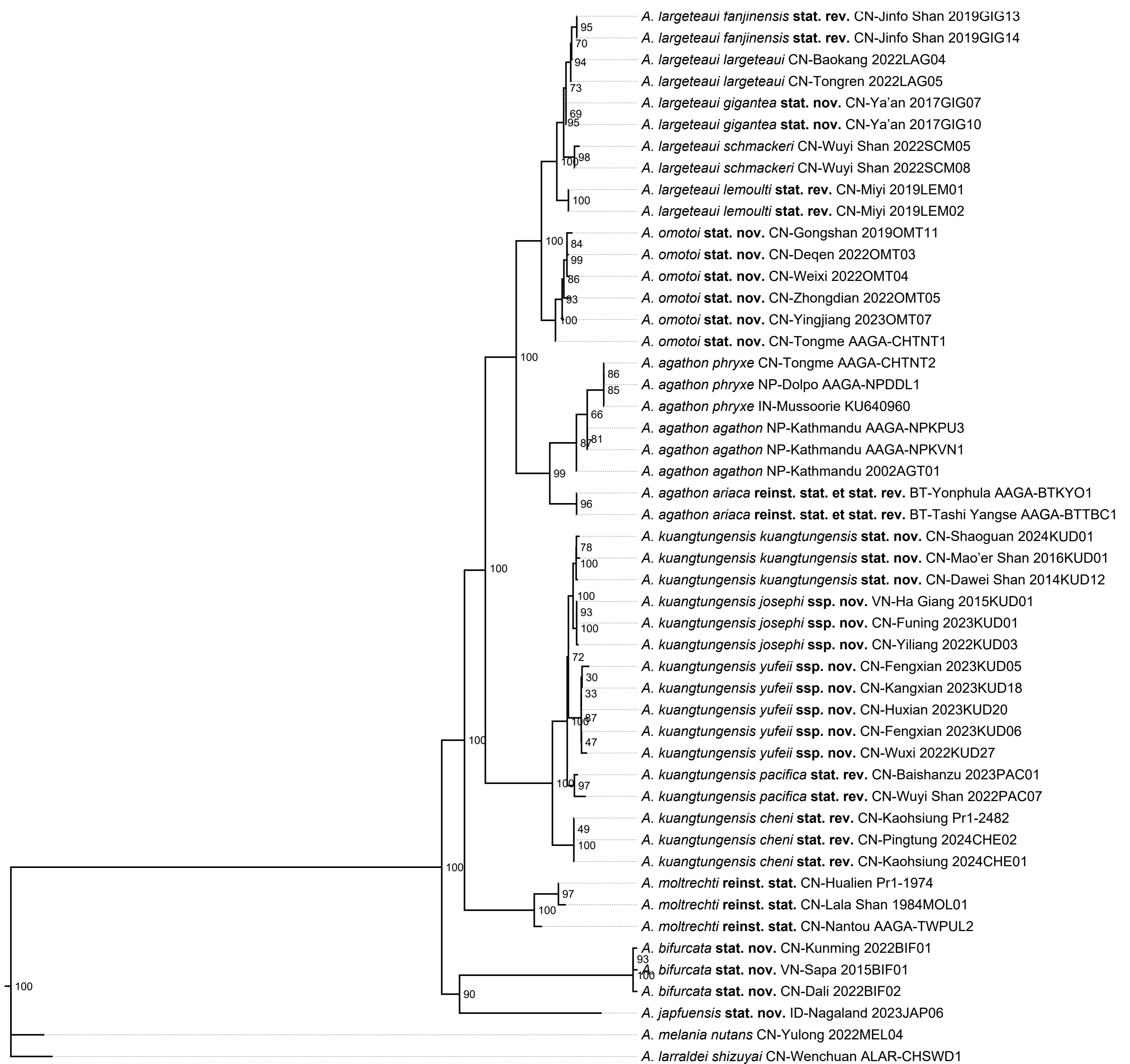

0.02

Supplement: Supplementary file 1 [file insects-15-00988-s001.zip › Suppl, Mat/Figure S2. The IQ-tree of all recognised taxa (nodevalue).pdf]
